# Rethinking Nonlinear Instrumental Variable Models through Prediction Validity

**Published:** 2022

**Authors:** Chunxiao Li, Cynthia Rudin, Tyler H. McCormick

**Affiliations:** Department of Statistical Science, Duke University, Durham, NC 27708, USA; Departments of Computer Science, Electrical and Computer Engineering, Statistical Science, Mathematics and Biostatistics & Bioinformatics, Duke University, Durham, NC 27708, USA; Department of Statistics and Department of Sociology, University of Washington, Seattle, WA 98195-4322, USA

**Keywords:** instrumental variables, causal inference, machine learning

## Abstract

Instrumental variables (IV) are widely used in the social and health sciences in situations where a researcher would like to measure a causal effect but cannot perform an experiment. For valid causal inference in an IV model, there must be external (exogenous) variation that (i) has a sufficiently large impact on the variable of interest (called the *relevance assumption*) and where (ii) the only pathway through which the external variation impacts the outcome is via the variable of interest (called the *exclusion restriction*). For statistical inference, researchers must also make assumptions about the functional form of the relationship between the three variables. Current practice assumes (i) and (ii) are met, then postulates a functional form with limited input from the data. In this paper, we describe a framework that leverages machine learning to validate these typically unchecked but consequential assumptions in the IV framework, providing the researcher empirical evidence about the quality of the instrument given the data at hand. Central to the proposed approach is the idea of *prediction validity*. Prediction validity checks that error terms – which should be independent from the instrument – cannot be modeled with machine learning any better than a model that is identically zero. We use prediction validity to develop both one-stage and two-stage approaches for IV, and demonstrate their performance on an example relevant to climate change policy.

## 1. Introduction

In many settings, particularly in the social and behavioral sciences, experiments are infeasible, impractical, or immoral. Without the artificial variation of an experiment, variables may interact in a way that makes it difficult to assess causal effects. Instrumental variable (IV) analysis is one approach to estimating causal effects when experiments are not possible (for an introduction see, for example, Didelez et al. 2010, Rosenbaum et al. 2010, Stock and Watson 2011, Glass et al. 2013, or Imbens 2014). In this paper, we describe a framework for estimating IV regression models that leverages machine learning to validate typically unchecked but consequential assumptions in the IV framework. Our work provides a necessary, though not sufficient, set of conditions of the IV model to be valid. Extending our method to also be a sufficient set of conditions would require information about unobserved variables, which we, of course, do not have.

To begin, consider the standard instrumental variables set up. First, we have the outcome of interest *Y*, which we believe could be causally explained by another variable, which we refer to as the treatment *T*. In an observational study, the causal impact of the treatment on the outcome of interest cannot be measured directly due to possible confounding or mediation. In the IV framework, we introduce an additional variable *Z*, (the instrument) that influences the outcome only through its effect on the treatment. The core intuition is that any variation in outcome *Y* that comes from variation in the treatment *T* due to variation in the instrument *Z* represents the causal impact of the treatment on the outcome. This causal impact is what the researcher aims to estimate.

Two critical assumptions underlie IV models. First, we assume that the association between the instrument and the treatment variable is nontrivial. That is, that the (exogenous) variation in the instrument leads to meaningful variation in the treatment variable, which means the variation is sufficiently strong so as to not be caused by noise. This is known as the *relevance assumption* (see Bound et al. 1995 for further discussion). The relevance assumption can be assessed using the observed instrument and treatment directly. Second, we must assume that the only source of variation in the outcome from the instrument is through changes in the treatment variable. This is known as the *exclusion restriction* (see Rosenzweig and Wolpin 2000, Angrist and Krueger 2001, or Angrist and Pischke 2010 for further discussion and contextual examples). In practice, the exclusion restriction cannot be verified with the data that a researcher has at hand. It is inherently a statement about unobserved variables (and if a researcher has access to a known confounder or mediator, after all, she could simply include it). However, this does not mean we must simply surrender and assume the exclusion restriction holds: we might be able to determine, for instance, whether it seems to be violated.

In the traditional IV setting, underlying both of these two core assumptions is the implicit notion of a correct functional form for these dependence pathways. For instance, in the traditional two stage least squares estimator, the first stage uses a *linear* model for treatment *T* based on observed covariates *X* and instrument *Z*, and the second stage uses a *linear* model for the outcome *Y* using *X* and the predicted values of treatment $\hat{T}$ from the first stage. Thus, in the traditional setting, both of the two critical assumptions use *correlation* as the key measurement. The relevance assumption in this context means that there exists a strong *correlation* between the instrument and the treatment. The exclusion restriction states that the instrument is not directly *correlated* to the outcomes, in other words, the instrument is not correlated to the error term in the second stage. *That is, the only relevant measure of dependence we would use for checking the exclusion restriction in the two stage least squares approach is linear (correlation)*. While previous work has used empirical data to evaluate the necessary conditions for the IV model assumptions (Rosenzweig and Wolpin, 2000), such work still focuses on a linear framework. These linearity assumptions are problematic when the dependencies are actually nonlinear.

To make IV analyses more powerful, recent work has extended IV to more complex machine learning models, including neural networks (Hartford et al., 2017; Adhikari et al., 2019; Singh et al., 2019; Guo and Small, 2016; Puli and Ranganath, 2020), however, it is unclear what the underlying assumptions are in these nonlinear models, and whether they hold for any given real problem.

In this work, we aim to precisely clarify assumptions underpinning the IV approach, and how they should change for nonlinear models. We address two questions. First, is it possible to propose a *general validity check for IV models that can be used no matter what the form of the model is*? Second, is it possible to *use this validity condition to guide the development of better models for IV*? We answer these two questions by proposing a unifying machine-learning-based perspective on IV models called *prediction validity.* The approach differs fundamentally from classical two stage least squares, whose results hold conditionally on the assumptions being satisfied and the functional form being known. In contrast, we present the entire problem in an optimization framework, where assumptions can be checked and empirically verified. In our framework, if a proposed instrument can predict the error terms, it does not appear to be a valid instrument. In particular, if the instrument can predict the error terms approximately as well as the function 0 (that is identically zero), then the proposed instrument passes the validity check; it cannot predict the error terms any better than the function 0 can. Because we use prediction error, rather than linear correlation, to check validity of the instrument, we are not restricted to linear models.

It is natural to assume is that if an instrument is actually valid, it should also appear to be. That is, a validity check on a valid instrument should always hold. As we will show, the new validity check always holds for linear models, whether the instrument is valid or not; this means that we could never have checked for the validity of instruments in the traditional two-stage-least-squares linear case. When we generalize to nonlinear models, the validity check does not always hold. Here, a traditional two stage approach would depend on some level of serendipity: it is entirely possible for an instrument to be (externally) known to be valid, but to violate an empirical validity check due to randomness in the data or fitting procedure. In that case, we show that changing the fitting procedure can fix such errors, so that instruments that are truly valid will also appear to be valid. We will show how to do this in our new two-stage and one-stage approach.

Thus, the methods in this paper provide necessary, but not sufficient conditions for evaluating the exclusion restriction. While our statistical framework is not able to completely verify the exclusion restriction, it provides the researcher empirical evidence about the quality of the instrument given the data at hand.

While our framework applies broadly, in this paper we focus on a powerful and flexible class of models, namely general additive models. These models are linear combinations of features, each of which is an arbitrarily nonlinear function of the input variables. For instance, this encompasses the types of generalized additive models that are powerful enough to yield results as accurate as neural networks for tabular data problems, even when dealing with complex data sets such as raw medical records (Caruana et al., 2015).

In the remainder of this section, we give some background on IV in practice, introduce the two stage least squares method, and define the two critical assumptions that use correlations as their key measurements. The organization of the rest of the paper is as follows:

- In Section 2, we generalize *the original assumptions of standard IV to the non-linear framework* and propose *a new empirical validity check*. The new versions of the two critical assumptions use loss functions to measure *predictability,* which provides a more general measurement of the relationship between two variables than (linear) correlation. The *empirical validity check* is defined to test the modified exclusion restriction and is applicable to the machine learning model framework.
- In Section 3, we propose a *new two-stage method for the general non-linear IV framework*, using prediction validity to check for valid instruments. This new two-stage method incorporates a constraint into the optimization problem to ensure that the instrument appears to be valid. If we have external knowledge that the instrument really is valid, the constraint should help to ensure valid inference. Alternatively, if the selected variable is not actually an instrument, the constraint can help us to determine that it is not an instrument.
- Also in Section 3, we present a *new one-stage procedure for IV analysis.* We incorporate both IV assumptions (relevance and exclusion) within a single mathematical framework by using one constraint to ensure that the instrument is strongly predictive of the treatment, satisfying the relevance assumption, and another to ensure the exclusion restriction, which is that the remainder is not able to be predicted by known variables. The one-stage approach has a possible advantage over the proposed two-stage approach in that it allows more flexibility for both constraints to be satisfied simultaneously.
- In Section 4, we provide a set of *feasibility proofs for the types of solutions available for our new two stage model under different model forms*. An interesting connection to the standard two-stage IV procedure appears in the case where both stages are linear models; here, the validity constraint is *always* satisfied, so that the traditional two stage model is the solution to the new two stage model. This shows a limitation of the standard approach: all proposed instruments appear to be valid instruments based on observed data, even if they are not. If we then allow the first stage to be more flexible, by using a general additive model (GAM), we show that the validity constraint is still satisfied. However, when the second stage is changed to a GAM, as we show, things change. If the second stage is a GAM and the first stage is a linear model, we might be able to identify that a variable is a non-instrument, but we would not be able to fix a situation where a variable appears to be a non-instrument when it is actually a valid instrument. If both stages are GAMs, we have the flexibility to fix an instrument that is valid but does not appear to be valid due to lack of serendipity in data or model fitting.
- In Section 5, we apply our two-stage and one-stage methods to simulated datasets. The simulation results that accord with theorems in Section 4 verify the theoretical results of our two stage method. We also show that our two stage method can efficiently *identify non-instruments*. We demonstrate that our two stage method achieves lower variance results than the traditional two-stage least squares method, and we show how one might fix a misspecified model in the presence of a valid instrument that appears to be invalid.
- In Section 6, we apply our two stage method to a real-world dataset on climate policy perspectives of voters who live near a wind-energy project. We show that our two stage method with more flexible model constructions does often outperform the traditional two-stage method in terms of more accurate estimation on the true causal effect.

In Section 8, we provide a summary of main ideas in this paper and point out directions for future work.

### Instrumental Variables in Practice.

Instrumental variables are widely used in natural experiments across multiple disciplines. In economics and the social sciences, IVs were originally used to estimate demand and supply curves (see Goldberger 1972 for further discussion), but have since been used far more widely. Common examples of IV designs include a policy change (e.g., a tax) that creates a change in a behavior that is the treatment of interest. Rainfall is commonly used as an instrument for changes in agriculture income. Rosenzweig and Wolpin (2000) provides an overview as well as a discussion of the critical role that models of human behavior play in evaluating IV models in such contexts. IV models are also frequently combined with experiments, as in the case of the encouragement design, which is common in public health. Let us say a researcher is interested in the impact of a treatment (say taking a flu vaccine) on the likelihood of getting the flu (see, e.g., Hirano et al. 2000). A regression that predicts likelihood of the flu based on whether or not a person got the vaccine will suffer from confounding. In the encouragement design, a randomly selected set of individuals receive a reminder or other prompting to get a flu shot. The encouragement produces variation in the probability that some people will get the flu shot, but since the encouragement is assigned randomly, it cannot be correlated with any other confounding variables. The variation in probability to get the flu shot that comes from being assigned the encouragement can then be used to identify the causal effect of getting a flu shot on getting the flu.

Genetic variation is also an increasingly common source of variation for IV models. Such models rely on Mendelian randomization, or the (random) assignment of genotypes at conception (Davey Smith and Ebrahim, 2003; Smith, 2007). Under a set of biological and statistical assumptions (see von Hinke et al. 2016; Didelez and Sheehan 2007; Didelez et al. 2010), associations between genotypes and the outcome of interest are unlikely to be due to any other biological or behavioral factors. Since the variation in genotypes is exogenous, this strategy yields an approach for understanding the causal influence of non-genetic risk factors on outcomes.

Let us commence with our review of the traditional approach to IV analysis.

### 1.1 Traditional Two Stage Method and Data Generation Processes

In this section, we discuss the typical two stage least squares setup for the IV model. We begin with some notation:

- Notation of Unknown Confounders and Mediators: *X_UN_* is the unknown confounder that influences both the treatment and the outcome of interest. It is the confounding effect we want to control for using the IV method. *X*_*UN,DEP*_ is the unknown mediator that influences both the instrument and the outcome of interest. It is what disqualifies a variable to be a valid instrument. ${\vec{X}}_{UN}$ and ${\vec{X}}_{UN,DEP}$ are the sample version of *X_UN_* and *X*_*UN,DEP*_ respectively.
- Notation of Sample Spaces: 𝒳 is the observed feature space, 𝒵 is the space of possible values for the instrument, 𝒯 is the space of possible values for the treatment, and 𝒴 are possible outcomes.
- Notation of Populations: Random variables are capitalized, whereas realizations are lower cases. For example, the observed covariates *X* is a random variable whose domain is 𝒳, whereas *x* is a realization of the observed covariates *X*. {*x, z, t, y*} are realizations of random variables {*X, Z, T, Y*} respectively.
- Notation of Samples: Samples are represented by matrices or vectors, whereas the *i*-th observation in a sample is a lower case with subscript *i*. For example, $\vec{X}$ is a sample of the observed covariates *X*, whereas *x_i_* is the *i*-th observation in the sample. {*x_i_, z_i_, t_i_, y_i_*} are the *i*-th observation of samples {$\vec{X}$, $\vec{Z}$, $\vec{t}$, $\vec{y}$} respectively. We generally use capital letters for matrices or random variables.
- Notation of Distributions: 𝒟_*X,Z*_ is a joint distribution over which *X* and *Z* are drawn. 𝒟_*X_UN_*_ is a distribution over which *X_UN_* is drawn. 𝒲_1_ and 𝒲_2_ are distributions of white noise that have zero mean, zero covariance and finite variance.
- Notation of Models: The data generation process for *T* and *Y* is defined using *f*_*ω*_true__ (*X*, *Z*) and *g*_*β*_true__ (*X*, *T*). Models developed using the data sample for predicting $\vec{t}$ and $\vec{y}$ are represented by $f_{\omega }(\vec{X},\vec{Z})$ and $g_{\beta }(\vec{X},\vec{t})$.

We use a running example of taxation rates (e.g., tax rates on cigarettes) as an instrumental variable $\vec{Z}$, smoking as a treatment $\vec{t}$, and health conditions as outcomes $\vec{y}$. Tax laws affect smoking (or cigarette consumption) through the price of cigarettes. Smoking has an influence on health conditions. Tax laws do not affect health conditions directly, they influence only the amount of smoking, which influences health conditions, given known covariates $\vec{X}$ and unknown confounders ${\vec{X}}_{UN}$. The data available are $\{x_{i},z_{i},t_{i},y_{i}{\}}_{i=1}^{n}$ containing the known covariates, values of the instrument, the treatment, and outcomes, respectively, for each individual (see Glass et al., 2013). Here, the unknown confounders ${\vec{X}}_{UN}$ that would impact both the amount of smoking and health outcomes provide the confounding effect that we need to control for and they are unobserved. For now, we assume there are no unknown mediators ${\vec{X}}_{UN,DEP}$ that would impact both the instrument and health outcomes directly.

In the context of instrumental variables, a popular form of estimation is known as two-stage-least-squares regression. In typical two-stage least squares regression, we would build linear models in Stage One and Stage Two respectively, and solve them using the method of least squares. In Stage One, we would build a linear model to predict the amount of smoking from tax law information and covariate information, $\hat{t}=f_{\hat{\omega }}(\vec{X},\vec{Z})=\langle \hat{\omega },[\vec{X},\vec{Z}]\rangle$, where $\vec{Z}$ is the value of the instrument (the presence of higher taxes), $\vec{X}$ contains known covariates (gender, age, etc.), and $\hat{\omega }$ is the estimated coefficient vector for the linear model. In Stage Two, we would estimate health conditions as a linear function of predicted amount of smoking and covariate information, $\hat{y}=g_{\hat{\beta }}(\vec{X},\hat{t})=\langle \hat{\beta },[\vec{X},\hat{t}]\rangle$, where $\hat{\beta }$ is the estimated coefficient of the linear model. This would provide an estimate for the effect of smoking on health conditions through the lens of the instrument. Since ${\vec{X}}_{UN}$ is not observed, it is not included in the estimation process. Thus, the fitted models of the traditional two-stage method are:

$$\hat{t}(\vec{X},\vec{Z})=f_{\hat{\omega }}(\vec{X},\vec{Z})\;\;\hat{y}(\vec{X},\hat{t})=g_{\hat{\beta }}(\vec{X},\hat{t}).$$

In what follows, we introduce two generative processes for data, one that contains an unobserved mediator and the other that does not. Let us consider the data as having been generated from one of these two processes.

#### Valid Instrument Data Generation Process.

**Figure 1:**
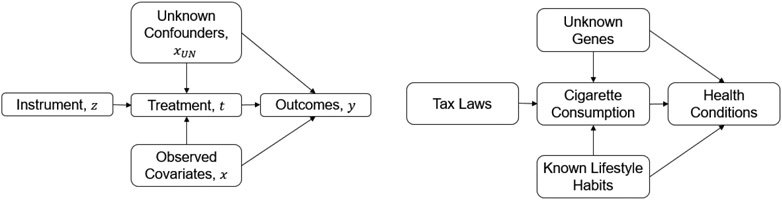
Causal graph without unobserved mediators (left) and concrete example (right). Data generation process for valid instruments.

First, in a setting where the IV assumptions are satisfied, we have

$$(X,Z)∼D_{X,Z}\;X_{UN}∼D_{X_{UN}},X⫫X_{UN}\;\text{and}\;Z⫫X_{UN}\;e_{1}∼W_{1},e_{2}∼W_{2}\;T=f_{\omega_{\text{true}}}(X,Z)+p(X_{UN})+e_{1}\;Y=g_{\beta_{\text{true}}}(X,T)+q(X_{UN})+e_{2}$$

where *f*_*ω*_true__ and *g*_*β*_true__ are functions of the known covariates *X* and the instrument *Z* in Stage One and Stage Two respectively, *p* and *q* are functions of the unknown confounder *X_UN_*, and *e*_1_ and *e*_2_ are random error terms. This is illustrated in Figure 1. Here, when a variable satisfies the IV assumptions, we call it a valid instrument.

Now consider the potential interference or inuence of outside unknown mediators *X_UN,DEP_* that would render the instrument invalid. In the tax laws example, if the same tax laws not only affect the price of the tobacco but also affect the price of some other unknown products, which affects other health-related consumption habits (this can be the consumption of alcohol, but we do not know) and further has an influence on health conditions, then the tax laws can also affect health conditions through other unknown approaches. As a result, if tax laws did affect health conditions, we cannot determine whether the effect followed from reducing cigarette consumption or other consumption habits. The causal graph containing the unknown mediators *X_UN,DEP_* is as follows:

#### Non-Instrument Data Generation Process.

**Figure 2:**
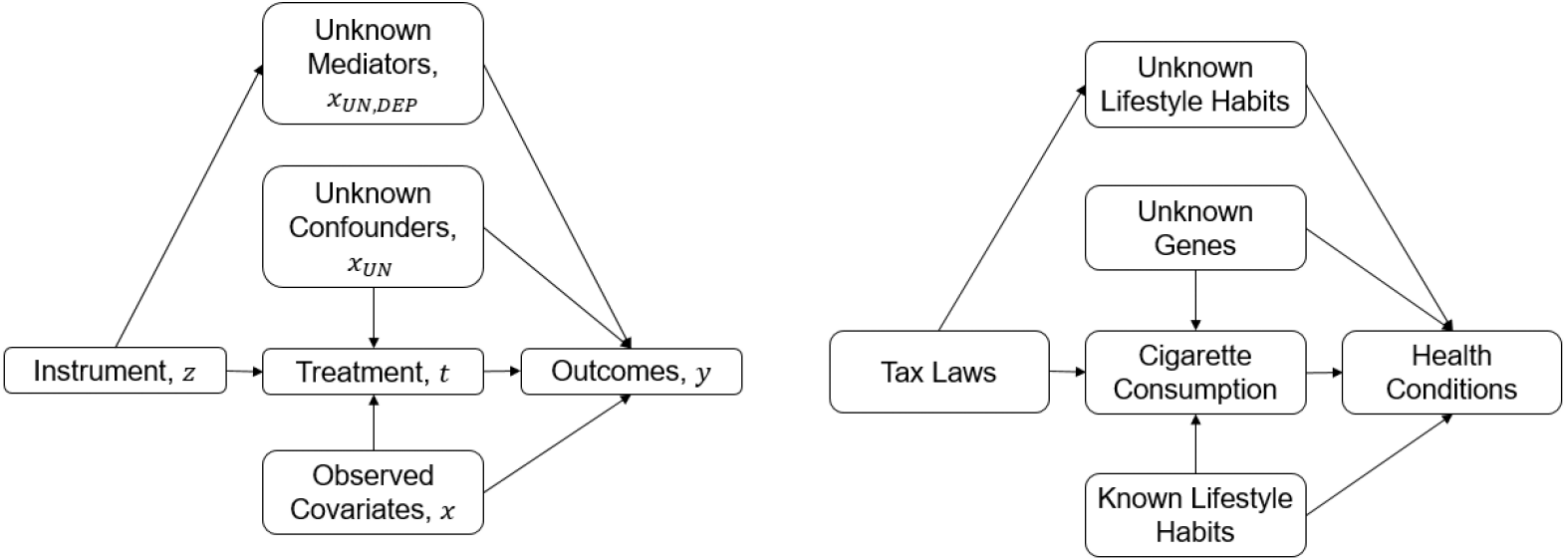
Graph with unobserved mediators (left) and concrete example (right).

$$(X,Z)∼D_{X,Z}\;X_{UN}∼D_{X_{UN}},X⫫X_{UN}\;\text{and}\;Z⫫X_{UN}\;e_{1}∼W_{1},e_{2}∼W_{2}\;X_{UN,DEP}≔X_{UN,DEP}(Z)\;T=f_{\omega_{\text{true}}}(X,Z)+p(X_{UN})+e_{1}\;Y=g_{\beta_{\text{true}}}(X,T)+q(X_{UN})+s(X_{UN,DEP})+e_{2}$$

where *s*(*X_UN,DEP_*) is a function of unknown mediators *X_UN,DEP_*, and *X_UN,DEP_* is a function of the instrument *Z*. Thus *s*(*X_UN,DEP_*) can also be written as a function of the instrument *Z*, *s*(*X_UN,DEP_*(*Z*)). Here the unknown mediators do not influence treatment, but they could. This data generation process is shown in Figure 2.

With these formal characterizations of valid instrument and non-instrument data generating processes, we can now formalize the two core assumptions. In an attempt to gesture towards an empirical test of these assumptions, we distinguish between the population and the sample instantiations of these two assumptions, using the notation of the generative models above and introducing new notations of true error terms in the first and second stage respectively.

*V* represents the true error term in the first stage, which is the remainder of *T* after removing the total causal effect of *X*, *Z* or their combination. That is,

$$V=T-f_{\omega_{\text{true}}}(X,Z),$$

where *f*_*ω*_true__ is the true model in the first stage. Also,

$$\vec{v}=\vec{t}-f_{\omega_{\text{true}}}(\vec{X},\vec{Z})$$

is the sample version of *V*. Note that we cannot calculate it because we do not know *f*_*ω*_true__. We will discuss that issue shortly. *U* represents the true error term in the second stage, which is the remainder of *Y* after removing the total causal effect of *X*, *T* or their combination. That is,

$$U=Y-g_{\beta_{\text{true}}}(X,T),$$

where *g*_*β*_true__ is the true model in the second stage.

$$\vec{u}=\vec{y}-g_{\beta_{\text{true}}}(\vec{X},\vec{t})$$

is the sample version of *U*. Again, we cannot calculate it because we do not know *g*_*β*_true__, and we will discuss that issue shortly.

##### Population Version of Relevance Assumption.

The instrument *Z* must be correlated with the treatment *T* in the first stage, *Cov*(*Z, T*) = *E*[(*Z* – *E*(*Z*))(*T* – *E*(*T*))] ≠ 0. Thus, the instrument is *relevant*.

##### Population Version of Exclusion Restriction.

The instrument *Z* must not be correlated with the true error term *U* in the second stage, *Cov*(*Z*, *U*) = *E*[(*Z* – *E*(*Z*))(*U* – *E*(*U*))] = 0.

##### Sample Version of Relevance Assumption.

The instrument $\vec{Z}$ cannot have all zero coefficients in the first stage. That is, for the model $\vec{t}=\omega_{1}\vec{X}+\omega_{2}\vec{Z}+\vec{v}$ in the first stage, at least one element in the vector *ω*_2_ is not zero. The sample version of the relevance assumption is assessed by the F-test with null hypothesis $H_{0}\;:\omega_{2}=\vec{0}$.

##### Sample Version of Exclusion Restriction.

The instrument $\vec{Z}$ must not be correlated with the true error term $\vec{u}$ in the second stage, i.e., $Cov(\vec{Z},\vec{u})\;=\;\frac{1}{n-1}\sum_{i=1}^{n}(z_{i}-\bar{z})(u_{i}-\bar{u})=0$. Note that since $\vec{u}$ is the true error term and is not observed, we would use an estimated error term $\hat{u}$ in practice to check this assumption, where $\hat{u}=\vec{y}-g_{\beta }(\vec{X},\hat{t})$, in which $\hat{t}$ is the estimated amount of treatment, and *g_β_* is constructed from data. $\hat{u}$ will not be the same as $\vec{u}$ because *X_UN_* and *X_UN,DEP_* are not known. For the same reason, *g_β_* is not the same as *g*_*β*_true__.

The exclusion restriction above implies that the only way the instrument *Z* affects outcomes *Y* is through the treatment *T* but not any other unknown mediators *X_UN,DEP_*. If there exist other unknown mediators *X_UN,DEP_* through which the instrument *Z* also has an influence on outcomes *Y*, then the exclusion restriction does not hold. In the example above, substitution between unhealthy behaviors would violate the exclusion restriction. For example, higher cigarette prices may drive consumers away from cigarettes, but if they instead buy other products that have negative health effects (e.g., alcohol), which is unmeasured, then the restriction is violated.

As mentioned previously, these assumptions are typically not tested using data but, rather, using domain knowledge (e.g., with a model of behavior in economics or understanding of biology in Mendelian randomization). In the next section we explicitly connect these assumptions, presented to this point in the two-stage-least-squares context, to a prediction model, generalizing these assumptions using the notion of prediction validity.

## 2. A machine learning perspective on the IV assumptions via prediction validity

Both assumptions for the traditional two-stage method rely on the linear framework, using linear correlation between variables. In the proposed framework, we will no longer use (linear) correlation and, instead propose using prediction loss. To determine how well several variables predict another variable, we consider how well they, together, can be used to predict it using functions of these variables from a pre-specified class. Using prediction rather than correlation expands the types of dependence that the procedure can capture and incorporates potential misspecification in the form of the model explicitly.

Using the prediction validity framework, we can derive our new modified relevance and exclusion assumptions. The new population versions and the new sample versions of the assumptions are given below:

### Prediction Validity Population Version of Relevance Assumption

This assumption concerns the relationship between *Z* and *T*. It states that *T* can be predicted better by *X* and *Z* together than by *X* alone.

$$\underset{f\in F}{\text{min}}\;loss(T,f(X,Z))\leq \underset{f\in F}{\text{min}}loss(T,f(X))-\epsilon ,$$

where 𝓕 is a class containing all possible models and *loss* is a non-negative loss function. Here, *ϵ* indicates a positive threshold. *ϵ* can be chosen as a small fraction of either one of the two loss terms. Written another way (that may seem unnecessarily complicated now but will be generalized later), the assumption is:

$$\;loss(T,f_{\omega }(X,Z))\leq loss(T,f_{\omega }(X))-\epsilon \;\;\text{where}\;f_{\omega }(X,Z)\in \text{arg}\;\underset{f\in F}{\text{min}}\;loss(T,f(X,Z)),\;\text{and similarly},\;f_{\omega }(X)\in \text{arg}\;\underset{f\in F}{\text{min}}\;loss(T,f(X)).$$

Here, *f_ω_* may not be identical to *f*_*ω*_true__ from the data generation process, because *X_UN_* is unknown.

This assumption, and all of the assumptions below, reduce to the standard correlation-based assumptions in the linear version of the problem. Thus, these are strict generalizations of the assumptions used in standard methodology.

### Prediction Validity Population Version of Exclusion Restriction

This assumption concerns the relationship between *U* and *Z*. It states that the instrument *Z* and known covariates *X* cannot be used to predict the true error term *U* any better than a model that is identically 0 can, with a predetermined tolerance *ϵ*′.

$$loss(U,h_{\alpha }(X,Z))\geq loss(U,0)-\epsilon^{\prime }\;\text{where}\;U=Y-g_{\beta_{\text{true}}}(X,T)\;\text{and}\;h_{\alpha }\in \text{arg}\;\underset{h\in H}{\text{min}}\;loss(U,h(X,Z))$$

where 𝓗 is a class containing all possible models and *loss* is a non-negative loss function. Here, *ϵ*′ indicates a positive threshold.

### Prediction Validity Sample Version of Relevance Assumption

This assumption concerns the relationship between $\vec{Z}$ and $\vec{t}$. It states that $\vec{t}$ can be predicted better by $\vec{X}$ and $\vec{Z}$ together than by $\vec{X}$ alone.

$$\underset{f\in F_{\text{class}}}{\text{min}}loss(\vec{t},f(\vec{X},\vec{Z}))\leq \underset{f\in F_{\text{class}}}{\text{min}}loss(\vec{t},f(\vec{X}))-\epsilon ,$$

or equivalently,

$$\;loss(\vec{t},f_{\omega }(\vec{X},\vec{Z}))\leq loss(\vec{t},f_{\omega }(\vec{X}))-\epsilon \;\;\text{where}\;f_{\omega }(\vec{X},\vec{Z})\in \text{arg}\;\underset{f\in F_{\text{class}}}{\text{min}}loss(\vec{t},f(\vec{X},\vec{Z}))\;\text{and similarly},\;f_{\omega }(\vec{X})\in \text{arg}\;\underset{f\in F_{\text{class}}}{\text{min}}loss(\vec{t},f(\vec{X})),$$

where *F*_class_ is a flexible class of models (where the usual reasonable measures have been taken to prevent overfitting). Here, *ϵ* indicates a positive threshold.

### Prediction Validity Sample Version of Exclusion Restriction

This assumption concerns the relationship between $\vec{u}$ and $\vec{Z}$. It states that the instrument $\vec{Z}$ and known covariates $\vec{X}$ cannot be used to predict the true error term $\vec{u}$ any better than a model that is identically 0 can, with a predetermined tolerance *ϵ*′.

$$loss(\vec{u},h_{\alpha }(\vec{X},\vec{Z}))\geq loss(\vec{u},0)-\epsilon^{\prime }\;\text{where}\;\vec{u}=\vec{y}-g_{\beta_{\text{true}}}(\vec{X},\vec{t})\;\text{and}\;h_{\alpha }\in {\text{argmin}}_{h\in H_{\text{class}}}loss(\vec{u},h(\vec{X},\vec{Z}))$$

where *H*_class_ is a flexible class of models (where the usual reasonable measures have been taken to prevent overfitting) and *loss* is a non-negative loss function. Here, *ϵ*′ indicates a positive threshold.

The prediction validity exclusion restriction states that no matter how hard we try to minimize the loss using the instrument *Z* and known covariates *X*, we still cannot achieve a loss lower than what we can achieve using the model that is identically 0. This assumption is true if the true error term, *U*, is independent from the instrument *Z* and known covariates *X*. However, if there exist unknown mediators *X_UN,DEP_* through which the instrument *Z* also has an influence on outcomes *Y*, then this exclusion restriction does not hold. In the case that the exclusion restriction does not hold, we should be able to observe empirically that fitting $\vec{u}$ using $\vec{Z}$ will lead to a lower loss. If that occurs, we should question the validity of *Z* as an instrument.

The sample versions are still not useful in practice since they rely on unmeasured quantities. We will focus on this in the next section.

### 2.1 Empirically verifying the prediction validity assumptions

In practice, with the instrument $\vec{Z}$, covariates $\vec{X}$ and the treatment $\vec{t}$ observable, the sample version of the relevance assumption can be assessed directly. However, due to the fact that the true error term $\vec{u}$ is never observable, the sample version of the exclusion restriction is not testable. Recall the formula of the true error term $\vec{u}=\vec{y}-g_{\beta_{\text{true}}}(\vec{X},\vec{t})$, where $g_{\beta_{\text{true}}}(\vec{X},\vec{t})$ is the true model in the second stage, which we do not have. To remove the influence of unmeasured confounders, both standard methods and our method use the predicted value of the treatment $\hat{t}$ (which uses the instrument) instead of the true treatment $\vec{t}$ in the second stage. Because $\hat{t}$ is estimated from $\vec{Z}$ and $\vec{X}$, it purposely excludes unmeasured confounders ${\vec{X}}_{UN}$. Then we would use the corresponding estimated error term $\hat{u}=\vec{y}-g_{\beta }(\vec{X},\hat{t})$ (where *g_β_* is learned from data) instead of the true error term $\vec{u}$.

In order to avoid using the double hat on *u* when we need to estimate $\hat{u}$ later, let us change notation, by defining the remainder $\vec{r}$ to be the estimated error term $\hat{u}$. We will separately estimate $\vec{r}$ using another machine learning model, and we call this estimate $\hat{r}$, also denoted by $h_{\alpha }(\vec{X},\vec{Z})$, which shows its dependence on $\vec{X}$ and $\vec{Z}$. Putting this together, in order to test the sample version of the exclusion restriction, we use the following empirical validity check.

#### Machine Learning Empirical Validity Check:

$$loss(\vec{r},\hat{r})\geq loss(\vec{r},0)-\epsilon^{\prime }\;\;(\text{can predict remainders no better than null model})\;\text{where}\;\vec{r}=\vec{y}-\hat{y},\;\;\hat{y}=g_{\beta }(\vec{X},\hat{t})\;\text{and}\;\hat{r}=h_{\alpha }(\vec{X},\vec{Z})\;\;(\text{remainder})\;\text{where}\;g_{\beta }\in {\text{argmin}}_{g\in G_{\text{class}}}loss(\vec{y},g(\vec{X},\hat{t}))\;\;(\text{modeled outcomes})\;\text{and}\;h_{\alpha }\in {\text{argmin}}_{h\in H_{\text{class}}}loss(\vec{r},h(\vec{X},\vec{Z}))\;\;(\text{modeled remainders})$$

where *G*_class_ and *H*_class_ are flexible classes of models (where the usual reasonable measures have been taken to prevent overfitting). Here, *ϵ*′ indicates a positive threshold. Here, *g_β_* may not be identical to *β*_*β*_true__ from the data generation process, because neither ${\vec{X}}_{UN}$ nor ${\vec{X}}_{UN,DEP}$ is known. Instead, *g_β_* contains all of the predictive strength of $\vec{X}$ and $\hat{t}$ for predicting $\vec{y}$.

The validity check can be used in two ways: posthoc, where the model is constructed first and the validity is checked afterwards, and second, where the model in the second stage is constrained so that it obeys the validity check. If the instrument is valid, the posthoc approach occasionally will mistakenly state that it is invalid, whereas the constrained approach will always yield an instrument that appears to be valid. If the proposed instrument is not an instrument, ideally both the posthoc and constrained approaches will reveal this information. If there is a feasible solution satisfying the constraints, then by definition *Z* is a feasible instrument. Therefore, we use the constrained approach in most of our experiments.

### 2.2 Connection to Adversarial Machine Learning

Our general machine learning validity connects to adversarial learning, in that in order for the ML empirical validity check to be valid, the remainders from the second stage must have been *generated* in a way that we cannot use them to *discriminate* between the outcomes any better than a model that is identically zero.

To turn this into an adversarial min/max formulation, one would maximize the loss for $loss(\vec{r},h_{\alpha }(\vec{X},\vec{Z}))$ with respect to $\vec{r}$. The discriminator, which consists of the optimization problem for *h*_*α*_, would aim to predict the remainders $\vec{r}$. If the generator wins, then it is not possible for us to predict $\vec{r}$ any better than 0 can. If the discriminator wins, then *h*_*α*_ can approximate $\vec{r}$ and the validity check fails.

The validity check is a feasibility condition, not an optimality condition. This is why the generator’s “max” does not appear, instead replaced by an inequality (in the first line of the validity check).

## 3. Optimization Methodology

To begin, we introduce the general additive model, which we will use to capture the non-linear dependence in our prediction validity framework.

**Definition** *A general additive model (GAM) is a general linear model that can be written as a linear combination of both linear and non-linear features, i.e., a general additive model with input variable X and target variable y has the following form:*

$$\hat{y}=b_{0}+b_{1}{\text{feature}}_{1}(X)+\cdots +b_{q}{\text{feature}}_{q}(X)$$

*where* feature_*j*_(*X*), *j* = 1, ⋯ , *q is a linear or non-linear function of X and X* = (*x*_1_, ⋯ , *x*_*p*_).

Note that the general additive model (GAM) defined above is different from the generalized additive model with the following form:

$$\hat{y}=b_{0}+b_{1}{\text{feature}}_{1}(x_{1})+\cdots +b_{p}{\text{feature}}_{p}(x_{p})$$

where feature_*j*_(*x_j_*), *j* = 1, ⋯ , *p* is a linear or non-linear function of *x_j_*.

Many of our calculations require least squares calculations, which means we need the following assumptions: There is no multi-collinearity (or perfect collinearity) between input variables, and the square matrix of the input variables is invertible (non-singular). This assumption holds for the remainder of this paper.

Consistency of nonlinear IV estimators is established by Newey and Powell (2003) via Lemma A1 and Theorem 4.1, under five assumptions: identifiability, approximability, smoothness, compactness, and continuity. Similar results also apply to Deep IV (Hartford et al., 2017).

### 3.1 Two Stage Method

We will first introduce the new two stage formulation, where the estimates for $\hat{t}$ and $\hat{y}$ are based on general loss minimization. In the general formulation, both stages can use nonlinear models. If using linear or general additive models (GAMs) in both stages, with the squared loss, the computations simplify and we can gain more insight. The empirical validity check is used as a constraint. Again, these reduce to the standard two-stage least squares case when the constraints are removed and when the loss in the formulation is the squared loss.

We present the one dimensional case here for exposition and, in the appendix, present the complete vectorized version along with a simplification.

#### Stage One

The optimization problem can be written as follows:

$$\omega \in \text{arg}\;\underset{\omega }{\text{min}}\;\sum_{i}loss(t_{i},{\hat{t}}_{i})\;\text{where}\;{\hat{t}}_{i}=f_{\omega }(x_{i},z_{i}).\;(\text{predict treatment})$$

This determines ${\hat{t}}_{i}=f_{\omega }(x_{i},z_{i})$ for Stage Two.

#### Stage Two


$$\;\beta \in \text{arg}\;\underset{\beta }{\text{min}}\;\sum_{i}loss(y_{i},{\hat{y}}_{i})\;\text{where}\;{\hat{y}}_{i}=g_{\beta }(x_{i},{\hat{t}}_{i})\;(\text{predict outcome})\;\;\text{s.t.}\;\;\beta \;\;\text{obeys}\;\sum_{i}loss(r_{i},{\hat{r}}_{i})\geq \sum_{i}loss(r_{i},0)-\epsilon^{\prime }\;\;(\text{the model cannot predict the remainder too much better than a zero model})\;\text{where}\;\;r_{i}\;=y_{i}-{\hat{y}}_{i}\;(\text{remainder})\;\;\text{and}\;\;{\hat{r}}_{i}\;=h_{\alpha }(x_{i},z_{i}),\;\text{where}\;\alpha \in \text{arg}\;\underset{\alpha }{\text{min}}\;\sum_{i}loss(r_{i},{\hat{r}}_{i})\;(\text{predict remainder}).$$

### 3.2 One Stage Method

While the proposed two-stage formulation can help us to answer the questions stated in the introduction, it is possible that the constraints may not be obeyed in the second stage because of a misspecified model in the first stage. Often there are many models that predict almost equally well on a finite dataset (see Semenova et al., 2022), and it is not clear exactly what the first stage model should be. It is possible that models that predict well in the first stage lead to residuals that can be predicted by the instrument in the second stage. When that happens, the experimenter is stuck – they have a valid instrument which appears to be invalid, with no mechanism to change it. The one stage formulation we will present next prevents this from happening. The formulation uses the notion of the “Rashomon set,” that is, the set of models for with loss less than *ϵ*.

The first stage is replaced with a constraint that says any model $\hat{t}$ is feasible if it predicts $\vec{t}$ well, that is, it is in the Rashomon set. This is equivalent in the Bayesian setting to forcing a high posterior for $\hat{t}$.

Similarly to the last subsection, we present the one dimensional setting for exposition and then present multiple dimensions in the Appendix.


$$\;\underset{\beta ,\omega }{\text{min}}\;\sum_{i}loss(y_{i},{\hat{y}}_{i})\;\text{where}\;{\hat{y}}_{i}=g_{\beta }(x_{i},{\hat{t}}_{i}),\;\text{and}\;{\hat{t}}_{i}=f_{\omega }(x_{i},z_{i})\;(\text{predict outcome})\;\;\text{s.t.}\;\;\omega \;\;\text{obeys}\;\sum_{i}loss(t_{i},{\hat{t}}_{i})\leq \sum_{i}loss(t_{i},f_{\omega }(x_{i}))-\epsilon \;\;(\text{the model can predict the treatment well enough so that it is in the Rashomon set})\;\;\text{and}\;\;\beta \;\;\text{obeys}\;\sum_{i}loss(r_{i},{\hat{r}}_{i})\geq \sum_{i}loss(r_{i},0)-\epsilon^{\prime }\;\;(\text{the model cannot predict the remainder too much better than a zero model})\;\text{where}\;\;r_{i}\;=y_{i}-{\hat{y}}_{i}\;(\text{remainder})\;\;\text{and}\;\;{\hat{r}}_{i}\;=h_{\alpha }(x_{i},z_{i}),\;\text{where}\;\alpha \in \text{arg}\;\underset{\alpha }{\text{min}}\;\sum_{i}loss(r_{i},{\hat{r}}_{i})\;(\text{predict remainder}).$$

### 3.3 Defining the Optimization Threshold *ϵ*′

In order to determine the optimization threshold *ϵ*′, this subsection introduces a new parameter *γ* to help with the calculation. Here, *γ* defines whether an estimate for the remainder $\vec{r}$ is sufficiently good. It compares that estimate with a baseline of all zero predictions. Once *γ* is defined, we can easily use it to calculate *ϵ*′ More specifically, here, *γ* is a relative loss, measured in percentages, whereas *ϵ*′ is absolute, measured in the units of the loss:

$$loss(\vec{r},\hat{r})=loss(\vec{r},0)-\epsilon^{\prime }\;\text{where}\;\epsilon^{\prime }≔\gamma \;loss(\tilde{r},0)\;\text{and}\;\tilde{r}\;\text{approximates}\;\vec{r},\;\text{so that the expression above yields}\;loss(\vec{r},\hat{r})\approx loss(\vec{r},0)-\gamma \;loss(\vec{r},0),\;\text{that is},\;\gamma \approx 1-\frac{loss(\vec{r},\hat{r})}{loss(\vec{r},0)}.$$

Note that here $\tilde{r}$ is different from $\hat{r}$. Here, $\tilde{r}$ is the remainder in the second stage from the traditional two stage method, and it has a closed form expression (please see more details in the appendix), while $\hat{r}$ is the estimation of the remainder in our two stage model. Since $\hat{r}$ is the prediction of the remainder of $\vec{r}$, $loss(\vec{r},\hat{r})$ is always smaller than $loss(\vec{r},0)$, but ideally it should not be too much smaller, assuming the instrument is valid. In fact, when the model choice is correctly specified and sufficiently fitted to the data so that $\hat{r}$ cannot predict $\vec{r}$ much better than a zero model, $loss(\vec{r},\hat{r})$ is close to $loss(\vec{r},0)$ and *γ* is small enough to be close to zero. However, when the model choice is not sufficiently complex, or when the selected variable *z* is not a valid instrument, then $loss(\vec{r},\hat{r})$ will be substantially smaller than $loss(\vec{r},0)$ and *γ* could be substantially greater than zero.

According to the definition of *γ*, $\gamma \approx 1-\frac{loss(\vec{r},\hat{r})}{loss(\vec{r},0)}$. If the models are linear models and the loss functions are squared loss, the influence of scale of the dataset in both loss functions will cancel each other out. However, for other model choice and loss function combinations, the scale of the dataset can be influential. In order to eliminate the influence of the scale, we normalize all the input features before modeling. Therefore, we need to choose *γ* as a function of only the sample size. The specific dependence between *γ* and the sample size can depend on the construction of the loss function. In our experiments, with sample size 1000, we choose *γ* = 1% as the percentage. (Simulated experiments are given near the beginning of Section 5 with different values of *γ*.)

## 4. Applicability for Our Two-Stage Method

In this section, we talk about the applicability of our two stage method. We discuss when it is helpful for identifying a valid instrument. We also discuss when it can help “fix” a valid instrument that appears to be invalid; in this case, the instrument only appears not to be valid because of misspecification of the model structure. As it turns out, the optimization results of our two stage model determine whether we may or may not be able to check the validity of the instrument, and further fix a seemingly invalid instrument.

We start with an overview of the optimization results in this section and then state each result formally.

### 4.1 Overview of Optimization Results

The tables below provide a summary of all possible optimization feasibility and satisfiability outcomes using our two stage method under different model constructions in the uni-dimensional (Table 2) and multi-dimensional (Table 3) cases of the instrument respectively, which illustrates the limitations and benefits of our two stage method under specific settings. These tables are a summary of our main theoretical results for the new IV framework involving prediction validity. The tables reference Theorem 1, Theorem 2 and Theorem 3 presented later in this section. Note that Theorem 2 is a special case of Theorem 1. By definition, an active constraint is met at equality. Table 1 provides an intuitive explanation of each potential optimization result using our two stage method, and is useful for understanding Tables 2 and 3.

The interesting aspect of Tables 2 and 3 is that their rows are different from each other; not all modeling choices yield the same set of possible results. Let us discuss the results in Table 2 for univariate instruments: In LM/LM and LM/GAM (top two rows), the constraints are *always* satisfied, and there is never an infeasible solution. This means these models will never appear to violate the validity condition, whether or not they are actually valid. Only the GAM/GAM combination permits checks and fixes to unsatisfied constraints; GAM/LM can only discover such problems but not fix them. For multivariate instruments, shown in Table 3, we can detect possible non-instruments in all cases, but can only potentially fix valid instruments when the second stage is nonlinear.

Before introducing any lemmas and theorems, we introduce the notation and settings that are used in this section. First, all the theorems in this section rely on the assumption that the flexible model classes for the treatment $\vec{t}$ and for the remainder $\vec{r}$ have the same level of complexity, that is, the predictor matrices for the treatment $\vec{t}$ and the remainder $\vec{r}$ are equivalent i.e., *X*_*t*_ = *X*_*r*_. Therefore, the hat matrix of the remainder $\vec{r}$, $H\;=\;X_{r}(X_{r}^{T}X_{r}{)}^{-1}X_{r}^{T}$ can be written as $H\;=\;X_{r}(X_{r}^{T}X_{r}{)}^{-1}X_{r}^{T}=X_{t}(X_{t}^{T}X_{t}{)}^{-1}X_{t}^{T}$, which is the hat matrix for the treatment $\vec{t}$. In the remainder of this section, we use the definition $H\;=\;X_{t}(X_{t}^{T}X_{t}{)}^{-1}X_{t}^{T}$ to represent both hat matrices for the remainder $\vec{r}$ and the treatment $\vec{t}$. The covariates are represented in an *n* × *p* matrix $\vec{X}$ whose column space is *p*–dimensional, i.e., $\vec{X}\;=\;(x_{1},\cdots ,x_{p})$. In the multi-dimensional instrument cases, the instrument $\vec{Z}$ is a *n* × *q* matrix whose column space is *q*–dimensional i.e. $\vec{Z}\;=\;(z_{1},\cdots ,z_{p})$. In the uni-dimensional cases, the instrument $\vec{Z}$ is a *n* × 1 matrix whose column space is 1–dimensional i.e. $\vec{Z}=(z_{1})$. To summarize, the notation is as follows for the traditional two-stage-least-squares setup:

#### Stage One

The predictor matrix of $\vec{t}$ in Stage One is $X_{t}=X_{t}(\vec{X},\vec{Z})$.

The hat matrix of $\vec{t}$ in Stage One is $H=X_{t}(X_{t}^{T}X_{t}{)}^{-1}X_{t}^{T}$.

The predicted value of $\vec{t}$ in Stage One is $\hat{t}\;=\;X_{t}\hat{\omega }$ and $\hat{\omega }\;=\;(X_{t}^{T}X_{t}{)}^{-1}X_{t}^{T}\vec{t}$. Thus, $\hat{t}\;=\;X_{t}\hat{\omega }=X_{t}(X_{t}^{T}X_{t}{)}^{-1}X_{t}^{T}\vec{t}\;=\;H\vec{t}$.

#### Stage Two

The predictor matrix of $\vec{y}$ in Stage Two is $X_{y}=X_{y}(\vec{X},\hat{t})$, where $\hat{t}=X_{t}\hat{\omega }$.

The hat matrix of $\vec{y}$ in Stage Two is $H_{1}\;=\;X_{y}(X_{y}^{T}X_{y}{)}^{-1}X_{y}^{T}$.

Appendix 8 provides these two stages formally in vector notation. For the squared loss, the objective (2) is quadratic and the constraint (3) is ellipsoidal.

### 4.2 Lemmas and Theorems for Instruments with More Than One Dimension

Let us provide some results where instruments are at least 2 dimensional. The covariates are (always) multi-dimensional. Note that the predictor matrix of $\vec{y}$ in the second stage, *X_y_* (see below), include the general additive model features (GAMs) when we are referring to the general cases or they contain only the linear terms when we are talking about the linear cases. All proofs for this section are in Appendix 8.

#### Linear Algebra Theorem

A triangular matrix is invertible, if and only if all of its diagonal entries are nonzero.

The statement of Lemma 1 refers to the multidimensional formulation of our two-stage model in Appendix 8, though we have restated all relevant material in the main text. For instance, Constraint (3) mentioned below is in the appendix.

**Lemma 1** *If the models in Stage One and Stage Two are both linear models, the optimal (minimum) solution of the objective function (2) (i.e.,* ${\hat{\beta }}_{\text{min}}\;=\;\text{arg}\;{\text{min}}_{\beta }\;\beta^{T}X_{y}^{T}X_{y}\beta -2\beta^{T}X_{y}^{T}\vec{y}+{\vec{y}}^{T}\vec{y}$*) equals the optimal (minimum) solution of the objective function on the left side of Constraint (3) (i.e.,* ${\hat{\beta }}_{\text{min}}^{\prime }\;=\;\text{arg}\;{\text{min}}_{\beta }\;\beta^{T}X_{y}^{T}HX_{y}\beta -2\beta^{T}X_{y}^{T}H\vec{y}+{\vec{y}}^{T}H\vec{y}$*), i.e.,* ${\hat{\beta }}_{\text{min}}={\hat{\beta }}_{\text{min}}^{\prime }$. *This statement is true regardless of the dimension of* $\vec{X}$ *and the dimension of* $\vec{Z}$.

The next two lemmas are extensions of Lemma 1. Lemma 2 extends to general additive models in the first stage, and Lemma 3 extends to additive models in both stages with specific forms.

**Lemma 2** *If the model in Stage One is a general additive model, and the model in Stage Two is a linear model, then the result of Lemma 1 still holds. This statement is true regardless of the dimension of* $\vec{X}$ *and the dimension of* $\vec{Z}$.

**Lemma 3** *If the models in Stage One and Stage Two are general additive models that have the following forms:*

$$\hat{t}=\omega_{1}{\text{feature}}_{1}(\vec{X})+\cdots +\omega_{k_{1}}{\text{feature}}_{k_{1}}(\vec{X})+\omega_{k_{1}+1}{\text{feature}}_{k_{1}+1}(\vec{X},\vec{Z})+\cdots +\;\;\omega_{k_{1}+k_{2}}{\text{feature}}_{k_{1}+k_{2}}(\vec{X},\vec{Z})$$

*where each* ${\text{feature}}_{j}(\vec{X})$, *j* = 1, ⋯ , *k*_1_ *is a linear or non-linear function of* $\vec{X}$ *and each* ${\text{feature}}_{j}(\vec{X},\vec{Z})$, *j* = *k*_1_ + 1, ⋯ , *k*_1_ + *k*_2_ *is a linear or non-linear function of* ($\vec{X}$, $\vec{Z}$) *or only* $\vec{Z}$*, and*

$$\hat{y}=\beta_{1}{\text{feature}}_{1}(\vec{X})+\cdots +\beta_{k_{1}^{\prime }}{\text{feature}}_{k_{1}^{\prime }}(\vec{X})+\beta_{k_{1}^{\prime }+1}\hat{t}$$

*where* $k_{1}^{\prime }\;\leq \;k_{1}$ *and* {$\{{\text{feature}}_{j}(\vec{X}){\}}_{j=1}^{k_{1}^{\prime }}$ *is a subset of* $\{{\text{feature}}_{j}(\vec{X}){\}}_{j=1}^{k_{1}}$*, then the result of Lemma 1 still holds. In other words, if the following conditions are satisfied:*

1. the input features of the covariates $\vec{X}$ in Stage Two are a subset of those in Stage One;
2. the model in Stage Two contains only the linear term of the predicted values of the treatment $\hat{t}$,

*then the result of Lemma 1 still holds. This statement is true regardless of the dimension of* $\vec{X}$ *and the dimension of* $\vec{Z}$.

Note that Lemma 3 is more general than both Lemma 1 and Lemma 2.

Our next result is interesting: it highlights a situation where one would expect the constraint to be sometimes satisfied at equality - but it cannot be. As it turns out, either the solution is infeasible (meaning that the constraint cannot be satisfied) or the constraint is irrelevant. We present a partial version of it that is easier to read, but provides only a special case, before providing the full version.

**Theorem 1 (Partial)** *If the model in Stage Two is a linear model, and if the model in Stage One is a linear model or a general additive model, either Constraint* (3) *(i.e.,* $\beta^{T}X_{y}^{T}HX_{y}\beta -2\beta^{T}X_{y}^{T}H\vec{y}+{\vec{y}}^{T}H\vec{y}\leq \epsilon^{\prime }$*) is not active or there is no feasible solution. This statement is true regardless of the dimension of* $\vec{X}$ *and the dimension of* $\vec{Z}$.

This theorem extends to more general cases, as we show next.

**Theorem 1 (Full)** *If the models in Stage One and Stage two are general additive models that have the following forms:*

$$\hat{t}=\omega_{1}{\text{feature}}_{1}(\vec{X})+\cdots +\omega_{k_{1}}{\text{feature}}_{k_{1}}(\vec{X})+\omega_{k_{1}+1}{\text{feature}}_{k_{1}+1}(\vec{X},\vec{Z})+\cdots +\;\;\omega_{k_{1}+k_{2}}{\text{feature}}_{k_{1}+k_{2}}(\vec{X},\vec{Z})$$

*where each* ${\text{feature}}_{j}(\vec{X})$, *j* = 1, ⋯ , *k*_1_ *is a linear or non-linear function of* $\vec{X}$ *and each* ${\text{feature}}_{j}(\vec{X},\vec{Z})$, *j* = *k*_1_ + 1, ⋯ , *k*_1_ + *k*_2_ *is a linear or non-linear function of* ($\vec{X}$, $\vec{Z}$) *or only* $\vec{Z}$*, and*

$$\hat{y}=\beta_{1}{\text{feature}}_{1}(\vec{X})+\cdots +\beta_{k_{1}^{\prime }}{\text{feature}}_{k_{1}^{\prime }}(\vec{X})+\beta_{k_{1}^{\prime }+1}\hat{t}$$

*where* $k_{1}^{\prime }\;\leq \;k_{1}$ *and* $\{{\text{feature}}_{j}(\vec{X}){\}}_{j=1}^{k_{1}^{\prime }}$ *is a subset of* $\{{\text{feature}}_{j}(\vec{X}){\}}_{j=1}^{k_{1}}$*, then either Constraint* (3) *(*$\beta^{T}X_{y}^{T}HX_{y}\beta -2\beta^{T}X_{y}^{T}H\vec{y}+{\vec{y}}^{T}H\vec{y}\leq \epsilon^{\prime }$) *is not active or there is no feasible solution. This statement is true regardless of the dimension of* $\vec{X}$ *and the dimension of* $\vec{Z}$. *In other words, if the following conditions are satisfied:*

1. the input features of the covariates $\vec{X}$ in Stage Two are a subset of those in Stage One;
2. the model in Stage Two contains only the linear term of the predicted values of the treatment $\hat{t}$,

*then either Constraint* (3) *(*$\beta^{T}X_{y}^{T}HX_{y}\beta -2\beta^{T}X_{y}^{T}H\vec{y}+{\vec{y}}^{T}H\vec{y}\leq \epsilon^{\prime }$*) is not active or there is no feasible solution*.

This finishes our results for higher-dimensional instruments for the two-stage approach. Let us work with one dimension now, where, as we prove below, under certain conditions, the constraint is not needed because it is never active. It is simply always satisfied.

### 4.3 Lemmas and Theorems for 1D Instruments

The case of a 1D instrument is a special case of the multi-dimensional case discussed above, but the range of potential optimization results is narrower than that of the multi-dimensional case, as we show in this section. Theorem 1’s conclusion is that the constraint is either not active or there is no feasible solution, whereas for Theorem 2 below, the conclusion is that the constraint is simply never active and always satisfied. This means that in practice, one never needs to check the constraint when using special cases for the Two-Stage method. In these cases, the solution would be the same whether or not we include this constraint (and we are back to standard practice by omitting it). In Theorem 2 partial and full versions, the second stage must have a linear dependence on the treatment effect $\hat{t}$. In order to derive a more general result where the second stage can depend nonlinearly on $\hat{t}$, we leverage two lemmas from linear algebra to derive Theorem 3 and its full version. Theorem 3 (Partial) applies when there is a linear first stage, and a second stage that can accommodate non-linear dependence on $\hat{t}$, whereas Theorem 3 (Full) handles general additive models in both stages, again permitting nonlinear dependence on $\hat{t}$ in the second stage. All proofs for this section are in Appendix 8.

**Theorem 2 (Partial)** *In the uni-dimensional case of the instrument* $\vec{Z}$*, if the models in Stage One and Stage Two are both linear models, Constraint* (3) *(i.e.,* $\beta^{T}X_{y}^{T}HX_{y}\beta -2\beta^{T}X_{y}^{T}H\vec{y}+{\vec{y}}^{T}H\vec{y}\leq \epsilon^{\prime }$*) is always satisfied and never active. This statement is true regardless of the dimension of* $\vec{X}$.

In the full version below, we allow additive models in both stages but in the second stage, the dependence on $\hat{t}$ is still linear.

**Theorem 2 (Full)** *In the uni-dimensional case of the instrument* $\vec{Z}$*, if the models in Stage One and Stage Two are general additive models that have the following forms:*

$$\hat{t}=\omega_{1}{\text{feature}}_{1}(\vec{X})+\cdots +\omega_{k}{\text{feature}}_{k}(\vec{X})+\omega_{k+1}{\text{feature}}_{k+1}(\vec{Z})$$

*where each* ${\text{feature}}_{j}(\vec{X})$, *j* = 1, ⋯ , *k* *is a linear or non-linear function of* $\vec{X}$ *and* ${\text{feature}}_{k+1}(\vec{Z})$ *is a linear or non-linear function of* $\vec{Z}$*, and*

$$\hat{y}=\beta_{1}{\text{feature}}_{1}(\vec{X})+\cdots +\beta_{k}{\text{feature}}_{k}(\vec{X})+\beta_{k+1}\hat{t}$$

*where each* ${\text{feature}}_{j}(\vec{X})$, *j* = 1, ⋯ , *k is a linear or non-linear function of* $\vec{X}$*, then Constraint* (3) *(i.e.,* $\beta^{T}X_{y}^{T}HX_{y}\beta -2\beta^{T}X_{y}^{T}H\vec{y}+{\vec{y}}^{T}H\vec{y}\leq \epsilon^{\prime }$*) is always satisfied (i.e., never active). This statement is true regardless of the dimension of* $\vec{X}$. *In other words, if the following conditions are satisfied:*

1. the models in Stage One and Stage two share the same input features of the covariates $\vec{X}$;
2. the model in Stage One contains only one input feature of the instrument $\vec{Z}$, which can be either linerar or non-linear;
3. the model in Stage One contains no interaction term of the covariates $\vec{X}$ and the instrument $\vec{Z}$;
4. the model in Stage Two only contains the linear term of the predicted values of the treatment $\hat{t}$,

*then Constraint* (3) *(i.e.,* $\beta^{T}X_{y}^{T}HX_{y}\beta -2\beta^{T}X_{y}^{T}H\vec{y}+{\vec{y}}^{T}H\vec{y}\leq \epsilon^{\prime }$*) is always satisfied and never active)*.

We would like to have more general dependence on $\hat{t}$ in the second stage. To do that, we leverage results from linear algebra in Lemma 4 and Lemma 5. These results allow us to prove that the constraint in always satisfied in cases where there is nonlinear dependence on $\hat{t}$ in the second stage. Theorem 3 has this result when the first stage is linear, and the full version of Theorem 3 has the result for when both stages are additive.

**Lemma 4** *If* $A\;\in \;R^{n\times m}$, *n* > *m is an upper trapezoidal matrix with non-zero diagonal entries, the matrix A*[*A^T^ A*]^−1^ *A^T^ is a block matrix of the form:*

$$A[A^{T}\;A{]}^{-1}A^{T}=\left(\begin{array}{cc} I_{m\times m} & 0_{m\times (n-m)}\\ 0_{(n-m)\times m} & 0_{\left(n-m)\times (n-m\right)} \end{array}\right),$$

*where I*_*m*×*m*_ *is an m-dimensional identity matrix*.

**Lemma 5** *If* $B\in R^{n\times n}$ *is an upper triangular matrix with non-zero diagonal entries, the matrix B*[*B^T^ B*]^−1^ *B^T^* = *I*_*n*×*n*_ *is an n-dimensional identity matrix*.

**Theorem 3 (Partial)** *In the uni-dimensional case for the instrument* $\vec{Z}$*, if the model in Stage One is a linear model, and the model in Stage Two is a general additive model, Constraint (3) (i.e.,* $\beta^{T}X_{y}^{T}HX_{y}\beta -2\beta^{T}X_{y}^{T}H\vec{y}+{\vec{y}}^{T}H\vec{y}\leq \epsilon^{\prime }$*) is never active. This statement is true regardless of the dimension of* $\vec{X}$.

**Theorem 3 (Full)** *In the uni-dimensional case for the instrument* $\vec{Z}$*, if models in Stage One and Stage Two are general additive models that have the following forms:*

$$\hat{t}=\omega_{1}{\text{feature}}_{1}(\vec{X})+\cdots +\omega_{k_{1}}{\text{feature}}_{k_{1}}(\vec{X})+\omega_{k_{1}+1}{\text{feature}}_{k_{1}+1}(\vec{Z})$$

*where each* ${\text{feature}}_{j}(\vec{X})$ *is a linear or non-linear function of* $\vec{X}$ *and* ${\text{feature}}_{k_{1}+1}(\vec{Z})$ *is a linear or non-linear function of* $\vec{Z}$ *and*

$$\hat{y}\;=\;\beta_{1}{\text{feature}}_{1}(\vec{X})+\cdots +\beta_{k_{1}}{\text{feature}}_{k_{1}}(\vec{X})+\beta_{k_{1}+1}\hat{t}+\beta_{k_{1}+2}{\text{feature}}_{k_{1}+1}(\vec{X},\hat{t})+\cdots \;\;+\beta_{k_{1}+k_{2}+1}{\text{feature}}_{k_{1}+k_{2}}(\vec{X},\hat{t})$$

*where each* ${\text{feature}}_{j}(\vec{X})$, *j* = 1, ⋯ , *k*_1_ *is a linear or non-linear function of* $\vec{X}$ *and* ${\text{feature}}_{j}(\vec{X},\hat{t})$, *j* = *k*_1_ + 1, ⋯ , *k*_1_ + *k*_2_ *is a non-linear function of* ($\vec{X}$, $\hat{t}$) *or a non-linear function of only* $\hat{t}$*, then Constraint* 3 *(*$\beta^{T}X_{y}^{T}HX_{y}\beta -2\beta^{T}X_{y}^{T}H\vec{y}+{\vec{y}}^{T}H\vec{y}\leq \epsilon^{\prime }$*) is never active. This statement is true regardless of the dimension of* $\vec{X}$. *In other words, the following conditions are satisfied:*

1. the models in Stage One and Stage Two are general additive models that satisfy all the conditions in Theorem 2.
2. the model in Stage Two contains non-linear features of the predicted values of the treatment $\hat{t}$ and the covariates $\vec{X}$,

*then Constraint* (3) *(*$\beta^{T}X_{y}^{T}HX_{y}\beta -2\beta^{T}X_{y}^{T}H\vec{y}+{\vec{y}}^{T}H\vec{y}\leq \epsilon^{\prime }$*) is never active*.

## 5. Simulation

In this section we perform simulation studies to examine the prediction validity framework. Section 5.1 verifies the theorems through simulation. Section 5.2 show the benefit of the one-stage method, namely that it can sometimes find a feasible solution when there is none in the two-stage approach. Section 5.3 is a case study on identifying whether a hypothesized instrument is not actually a valid instrument. Section 5.4 shows that our two stage method’s confidence intervals on the true treatment effect estimate tend to be wider than those of the traditional method, which indicates that our new method can provide more robust confidence intervals on the true treatment effect. An interesting result in Section 5.5 shows that if we have a valid instrument but a misspecified model, our method can detect and fix the misspecified model.

### 5.1 Verifying Theorems

#### Simulations Verifying Theorem 1:

Theorem 1 states that, in settings with multidimensional instruments, if the prediction model in the first stage is a linear or general additive model and the prediction model in the second stage is a linear model, either the constraint is not active or there is no feasible solution. In order to verify this result experimentally, we conduct a series of simulations with different data generation processes, prediction models, and types of error terms.

The simulation results presented in Table 10 in the appendix accord with Theorem 1. The simulation results show that Theorem 1 is true when the prediction model form is the same as that of the generation model. The resuls also show that Theorem 1 holds as long as the prediction models satisfy their conditions, regardless of the form of the data generation functions. Other cases in Theorem 1 (Full) can also be verified by similar simulations.

#### Simulations Verifying Theorem 2 and Theorem 3:

These theorems state that, in the uni-dimensional case for the instrument $\vec{Z}$, if the prediction model in the first stage is a linear model and the prediction model in the second stage is a linear or general additive model, the constraint is never active.

The simulation results presented in Table 9 in the appendix accord with Theorem 2 and Theorem 3. The simulations empirically verify Theorem 2 and Theorem 3, regardless of the type of the error term. It is also shown that Theorem 2 and Theorem 3 hold as long as the prediction models satisfy their conditions, regardless of the forms of the data-generation functions. As with the previous theorem, other cases in the full versions of Theorem 2 and Theorem 3 can also be verified by similar simulations.

### 5.2 Simulations with One Stage Method

The one-stage method is not limited by the restrictions on model form given within Theorem 1, Theorem 2, and Theorem 3. In particular, the one-stage procedure can provide more flexibility in the optimization process. We demonstrate this advantage by comparing its results with those of the two-stage method through simulations. The simulation results are presented in Table 11 in the appendix. Our general two-stage method and one-stage method perform equally well when both of the two models are general additive models. When the second stage is linear, our one-stage method is not subject to Theorem 1 and *can outperform our general two-stage method. The two-stage method cannot provide a feasible solution in some cases where the one-stage method has a feasible solution*. This is particularly useful when the two-stage method indicates that a valid instrument is not an instrument; there is no easy remedy besides the one-stage method.

Note that the conditions under which Theorem 2 and Theorem 3 apply also do not restrict the model form of our one-stage method.

### 5.3 Identifying Non-Instruments and Valid Instruments

In the simulations below, we assume the correct model form; that is, we assume we had made modeling choices with sufficient complexity so that our model could potentially represent ground truth.

#### Simulation Setup:

In what follows, we assume we correctly specified the model form in the first stage, but that there can be unknown mediators in the second stage. We construct 1000 simulations for both the valid instrument case and the non-instrument case respectively. We use the following data generation mechanism.

Data Generation (Construct Valid Instrument):

$$t\;=\;x+z+z^{2}+e_{1}\;y\;=\;x+t+e_{2}.$$

Data Generation (Construct Non-Instrument):

$$x_{UN,DEP}\;=\;z^{3}\;\;t\;=\;x+z+z^{2}+e_{1}\;\;y\;=\;x+\phi \cdot x_{UN,DEP}+t+e_{2}.$$

where *x* ~ *N*(0, 1), *z* ~ *N*(0, 1) and $\left(\begin{array}{c} e_{1}\\ e_{2} \end{array}\right)∼N\left(\left(\begin{array}{c} 0\\ 0 \end{array}\right),\left(\begin{array}{cc} 1 & 0.8\\ 0.8 & 1 \end{array}\right)\right)$.

Here, *z* is not a valid instrument because there is a mediator *x_UN,DEP_* that will be an unknown function of *z*. The parameter *ϕ* is used to quantify the strength of influence from the unknown mediator on the “validity” of the instrument. The correlation between *e*_1_ and *e*_2_ introduces unmeasured confounding: these correlated variables impact both the treatment and outcome.

Here, we will use the same form of model for the prediction process. Thus, the predictor matrices used in the three prediction models for the treatment *t*, outcomes *y* and the remainder *r* are as follows.


$$X_{t}\;=\;(x,z,z^{2})\;X_{y}\;=\;(x,\hat{t})\;X_{r}\;=\;(x,z,z^{2}).$$

#### Two Stage Model Validity Results:

We show that our two stage method can identify when the proposed instrument is valid and when it is not. To do this, we ran 2000 simulations, where 1000 of them used a valid instrument, and 1000 of them used a non-instrument. Table 4 shows that our two-stage method identified whether the instrument is valid in each of the simulations.

When we set *ϕ* = 1 and *γ* = 1%, then the accuracies are 100%. This indicates that the valid instruments and non-instruments are perfectly separated when the unmeasured mediators have a fairly strong influence. However, if we set *ϕ* = 0.1 and *γ* = 0.1%, the accuracies drop. The results are shown in Table 5. It shows that our two stage method can still identify the valid instruments and non-instruments well, even though the unmeasured mediator is weak. When the strength of the unmeasured mediator is weak, non-instruments are more likely to pass a validity test.

### 5.4 Robustness of ML-IV

This section shows that when the instrument is valid, the coefficients obtained by our new method are more stable than those obtained by the traditional two-stage-least-squares method, if even we only use a class of polynomial models as prediction models, when the ground truth is not necessarily polynomial. We adopt some similar data generation models and metrics as the full matching approach of Kang et al. (2016). In this paper, we use the following model for the data generation process.

$$t\;=\;x+\pi f(z)+e_{1}\;y\;=\;x+\beta t+e_{2}$$

where *x* ~ *N*(0, 1), *z* ~ *N*(0, 1) and $\left(\begin{array}{c} e_{1}\\ e_{2} \end{array}\right)∼N\left(\left(\begin{array}{c} 0\\ 0 \end{array}\right),\left(\begin{array}{cc} 1 & 0.8\\ 0.8 & 1 \end{array}\right)\right)$. The *f*(·) is a predetermined non-linear function of the instrument *z*. The parameter *β* in the second stage is fixed to be 1, while the parameter *π* in the first stage can be varied. We use the value of the parameter *π* to quantify the strength of the instrument *z*. We consider the following functions *f*(·) of the instrument z and the corresponding predictor matrices for the treatment *t*:

- Quadratic function: *f*(*z*) = *z*^2^ and *X_t_* = (*x*, *z*, *z*^2^);
- Cubic function: *f*(*z*) = *z*^3^ and *X_t_* = (*x*, *z*, *z*^2^, *z*^3^);
- Exponential function: *f*(*z*) = exp(*z*) and *X_t_* = (*x*, *z*, *z*^2^, *z*^3^)
- Log function: *f*(*z*) = log(∣*z*∣) and *X_t_* = (*x*, *z*, *z*^2^, *z*^3^)
- Square root function: $f(z)=\sqrt{|z|}$ and *X_t_* = (*x*, *z*, *z*^2^, *z*^3^)
- Logistic function: $f(z)=\frac{1}{1+\text{exp}(-z)}$ and *X_t_* = (*x*, *z*, *z*^2^, *z*^3^)

**Figure 3:**
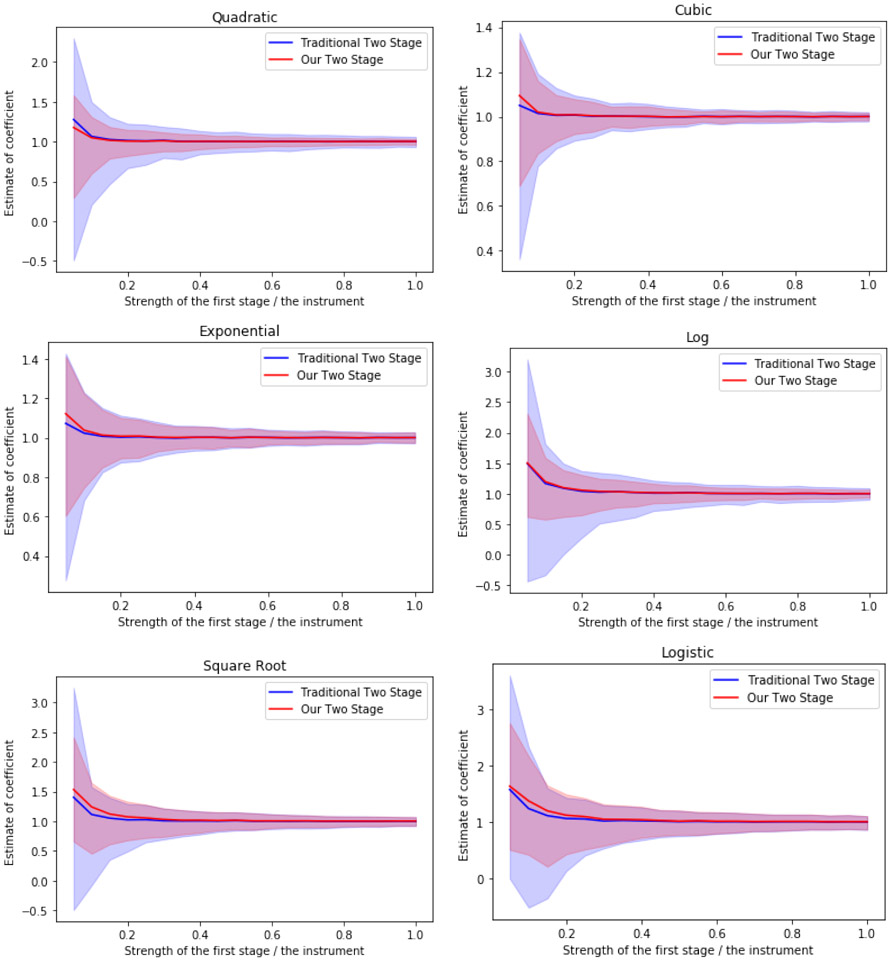
Our two stage method versus the traditional two-stage-least-squares method

For each functional form, we vary the parameter *π* from 0.1 to 1 by 0.05. For each unique combination of *f*(·) and *π*, we simulate the process 1000 times and display the estimates of the coefficient *β*. In Figure 3, the blue line represents the median estimate of the parameter *β* by the traditional two stage method, while the red line represents the median estimate of the parameter *β* by our two stage method. The blue region represents the 95% confidence interval of the parameter *β* by the traditional two stage method, while the red line represents the 95% confidence interval of the parameter *β* by our two stage method. The decreasing lines show that as the first stage become stronger, the estimation of the parameter *β* is closer to 1, or in other words, the estimation of the true causal effect becomes more accurate. *The fact that the blue region is wider than the red region shows that our two stage method provides more robust estimation than the traditional two-stage-least-squares method*.

### 5.5 Detect and address misspecification

#### Example 1: When model misspecification can be addressed.

In the following example, we have a valid instrument but a misspecified model, in that the data generation is a quadratic model, but the prediction model is linear. If we use a quadratic prediction model for the remainder *r*, we can detect that the model was misspecified. In that case, we can fix the modeling choice for the treatment $\hat{t}$ so that the instrument appears to be valid.

In particular, we use the following data generation process to construct a valid instrument case:

$$t\;=\;x+z+z^{2}+e_{1}\;y\;=\;x+t+e_{2},$$

where *x* ~ *N*(0, 1), *z* ~ *N*(0, 1) and $\left(\begin{array}{c} e_{1}\\ e_{2} \end{array}\right)∼N\left(\left(\begin{array}{c} 0\\ 0 \end{array}\right),\left(\begin{array}{cc} 1 & 0.8\\ 0.8 & 1 \end{array}\right)\right)$. Again, the correlation between *e*_1_ and *e*_2_ introduces unmeasured confounding, since these correlated variables impact both the treatment and outcome.

Here are the predictor matrices used in the three prediction models for the treatment *t*, outcomes *y* and the remainder *r* respectively:

$$X_{t}\;=\;(x,z)\;X_{y}\;=\;(x,\hat{t})\;X_{r}\;=\;(x,z,z^{2}).$$

Here we use a more flexible class of prediction models for the remainder *r* than that for the treatment *t*, which helps us to potentially detect a misspecified model when the instrument is actually valid. In this case, the quadratic model form for the remainder *r* can detect the quadratic dependence on the instrument *z*, which was not detected by the linear model for the treatment *t* in the first stage: since the remainder can be well-predicted by a more flexible model class, the loss between *r* and its estimated value $\hat{r}$ is significantly smaller than the loss between *r* and zero. As a result, the modeller would be able to conclude that the constraint is not satisfied due to a misspecified model.

If we then apply the quadratic model form in the first stage for estimation of the treatment *t*, the constraint can be satisfied again and the misspecified model is fixed. The predictor matrices used in the fixed modeling process for the treatment *t*, outcomes *y* and the remainder *r* are respectively:

$$X_{t}\;=\;(x,z,z^{2})\;X_{y}\;=\;(x,\hat{t})\;X_{r}\;=\;(x,z,z^{2},z^{3}).$$

The values of loss functions on both sides of the constraint, *loss*(*r*, $\hat{r}$) and *loss*(*r*, 0) for the two modeling choices (insufficient and sufficient) are as follows.

The results in Table 6 show that if we choose percentage *γ* equal to 10%, then our misspecified model (the linear first stage) will be rejected while the (correct) quadratic first stage will be accepted.

In the example above, we demonstrated than a misspecified model can be detected and fixed, whereas the following example shows a non-instrument that cannot be fixed.

#### Example 2: Attempts to fix a non-instrument will be unsuccessful.

Let us now switch to working with the non-instrument case from in Section 5.3, where *t* = *x*+*z*+*z*^2^+*e*_1_, *y* = *x* + *x_UN,DEP_* + *t* + *e*_2_ and *x_UN,DEP_* = *z*^3^. In Section 5.3, we used the following predictor matrices for the treatment *t*, outcomes *y* and the remainder *r* respectively:

$$X_{t}\;=\;(x,z,z^{2})\;X_{y}\;=\;(x,\hat{t})\;X_{r}\;=\;(x,z,z^{2},z^{3}).$$

If we had tested this setup, our validity constraint would have been violated and the proposed instrument would (correctly) appear invalid. If we attempt to fix the proposed instrument, we can add a more complicated dependence of the model $\hat{t}$ on the instrument *z*. (We cannot include a term that depends directly on *z* in the model for $\hat{y}$ because that would violate the definition of a valid instrument.) In this case, we add a cubic term *z*^3^ to the predictor matrix for the treatment *t*. When we do that, we also would like the model for the remainder $\hat{r}$ to be more flexible in order to detect possible misspecified models, so we add an additional *z*^4^ term to the remainder model. The predictor matrices used for new modeling choices of the treatment *t*, outcomes *y* and the remainder *r* are now respectively:

$$X_{t}\;=\;(x,z,z^{2},z^{3})\;X_{y}\;=\;(x,\hat{t})\;X_{r}\;=\;(x,z,z^{2},z^{3},z^{4}).$$

The values of the loss functions on both sides of the constraint, *loss*(*r*, $\hat{r}$) and *loss*(*r*, 0) for these two modeling choices are as follows. As a reminder, both modeling choices are reasonable, but neither can fix the non-instrument.

The results in Table 7 show that the attempt to fix the non-instrument by adding the cubic term was not successful: the true percentage for the second modeling choice is no better than that of the first model.

## 6. Example: Backlash against climate change

In this section, we tested our two-stage and one-stage methods on data from the paper entitled *Electoral Backlash Against Climate Policy: A Natural Experiment on Retrospective Voting and Local Resistance to Public Policy* by Stokes (2016). We replicated the traditional two-stage least squares regression form within the paper, and applied our two-stage and one-stage methods.

Here, we give a brief introduction of this paper and its data. This paper investigates whether living close to a wind energy project leads citizens or residents to vote against an incumbent government due to its climate policy. The data consists of the election, census and wind energy project data of 708 valid precincts in Ontario, Canada. Each row represents a valid precinct. For each precinct, the dataset includes: the average wind power (log) in a precinct as the instrument *z*, whether there is a proposed wind turbine within 3 km of the precinct in 2011 as the treatment *t*, the change in the Liberal Party vote share in that precinct between the 2007 and 2011 elections as outcomes *y*, as well as features about geographical information as other covariates *x*.

Due to the fact that the treatment *t* is a binary variable, we apply the kernel version two-stage ML-IV method (see appendix A) and use logistic regression for the binary variable in the first stage. Since there is no closed-form solution for the coefficients of the logistic regression, we use the general loss version of our methods instead of the square loss version.

**Figure 4:**
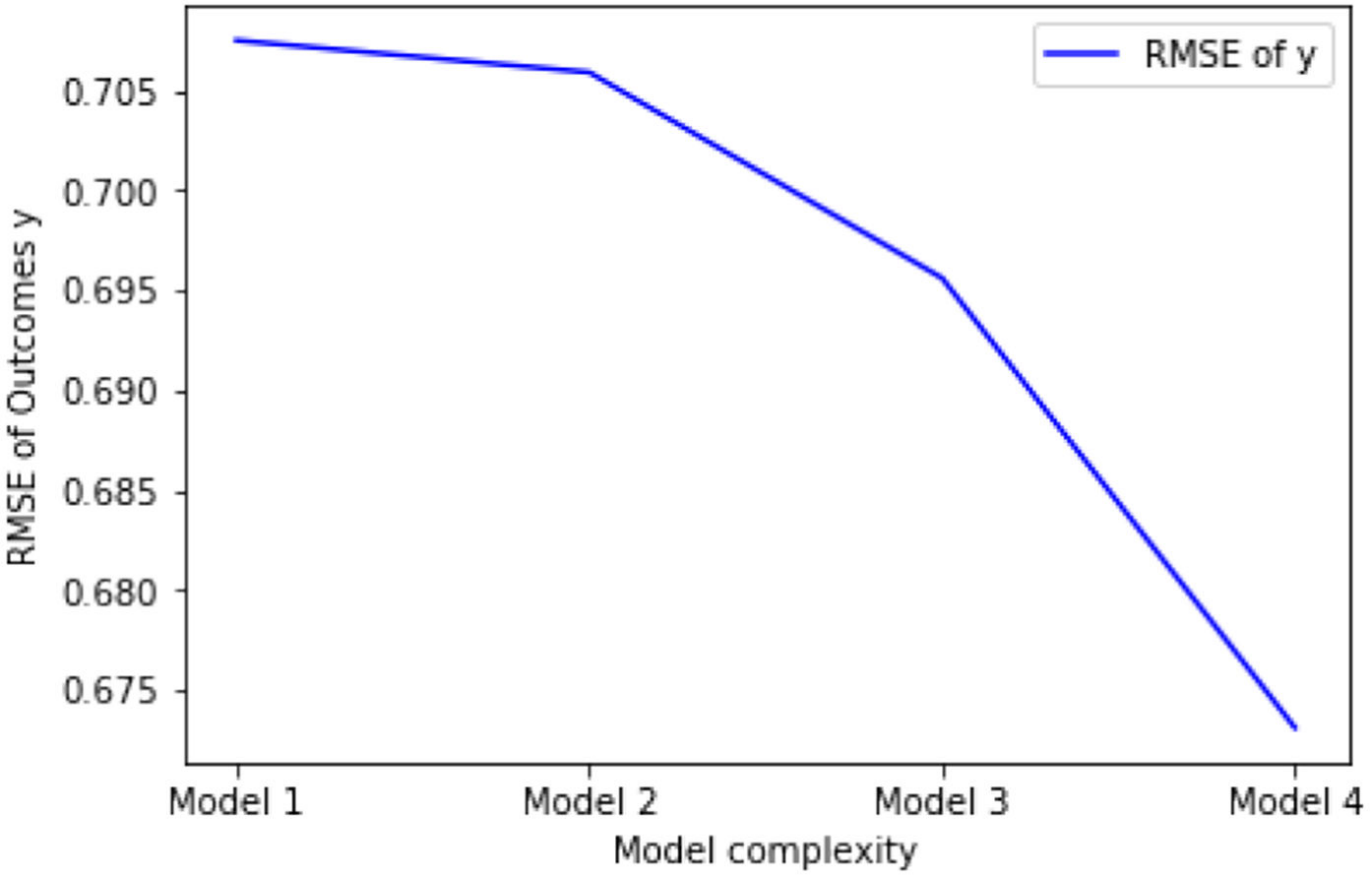
RMSE of outcomes versus models with different model complexity

**Figure 5:**
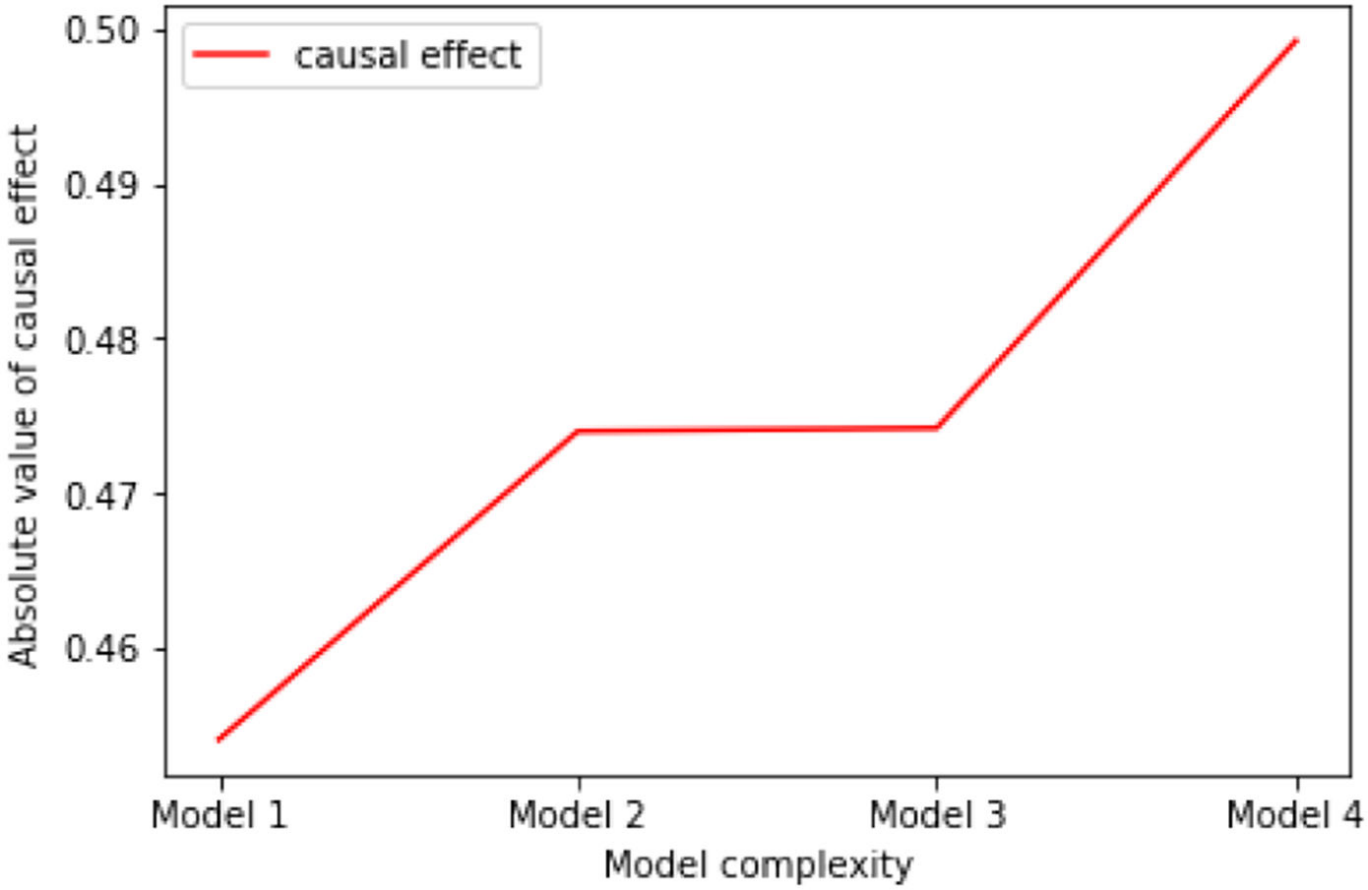
Causal effect size versus models with different model complexity

The data analysis procedures are as follows. First, we checked the relevance assumption and the exclusion restriction of the instrument *z* in this dataset. In particular, since the input features containing the instrument *z* contribute to the first stage model, the relevance assumption is satisfied and the instrument *z* has a strong first stage. Since our empirical validity check passed, the exclusion restriction is also satisfied and the instrument *z* appears valid. Second, we built four different models using different input features and used RMSE on the outcome as the metric to compare prediction performance. In the modeling process, 10-fold cross validation was used to ensure the stability of our results. The results are shown in Table 8, and the important parts of this table are plotted in Figures 4 and 5. Note that we have normalized all input variables when pre-processing, which means the results shown in the table are standardized.

According to Figures 4 and 5, we can draw the following two conclusions. First, as we choose more complicated models, our methods can provide more accurate prediction of outcomes than the traditional two-stage-least-squares method. This indicates that more complicated relationships between the variables may exist, and are taken into account by our approach. Second, as prediction power increases, the causal effect size also increases. This implies that the traditional two-stage method may underestimate the true causal effect for this important dataset by using a simple linear framework, while our methods may be able to provide more accurate estimation.

## 7. Recommendations for practice

In this section we provide concrete recommendations for scientists using IV models to address substantive questions. As mentioned previously, these recommendations assure necessary but not sufficient conditions and, thus, augment, rather than replace, the current state of practice. We provide guidance on the uni-dimensional two-stage case, following Table 2, however practitioners with multivariate instruments should consult Table 3. That is, in this section, the scientist has a single proposed instrumental variable, and would like to use the 2-stage case.

First, the scientist should decide whether a linear model is sufficient for their dataset. One can check this in both the first and second stage using both linear models and nonlinear models to determine whether the nonlinear terms improve prediction accuracy without overfitting.

If a linear model in both stages is sufficient, then the first step is to check the relevance assumption. To do this, one models the treatment as both a function of the covariates and the proposed instrument, and then again as a function of just the covariates; if the first of these two models is better than the second, the relevance assumption is satisfied. There is no need to check the exclusion restriction, since it will always appear satisfied based on observable variables (though this, of course, does not address unmeasured confounding or mediation).

If a linear model in the first stage is sufficient but one chooses an additive model in the second stage, again, one should check the relevance assumption as described in the previous paragraph, but there is no need to check the exclusion restriction, since it will always appear satisfied based on observable confounds (again, this does not address unmeasured confounding or mediation).

If a linear model in the first stage is not sufficient, a general additive model could be developed for the first stage. Again, one should check the relevance assumption is satisfied by determining that a model that includes the proposed instrument predicts the treatment better than one without the proposed instrument. Then, the scientist should decide whether a linear model is sufficient in the second stage, and at that point we can check the exclusion restriction.

When the first stage is a GAM and the second stage is linear, following the procedure in Section 3.1, whose math is in Section 8, according to Table 2, we would find that either there is a feasible solution where the constraint is not active, or there is no feasible solution (this is by Theorem 1). In the case of a feasible solution, the proposed instrument passes the check. In the second case, it fails. It is then possible that the proposed instrument is not valid.

When both the first and second stages are chosen to be GAMs, either 1) there is a feasible solution and the constraint may or may not be active, in which case the proposed instrument passes the check, or 2) there is no feasible solution, in which case it does not pass the check. In the case where the constraint is active, that is when the a posthoc approach (doing the traditional two stage without constraints and then checking them afterwards) would have made the instrument appear to be invalid, even though it might actually be valid; our two-stage procedure fixed the problem.

The models from this two-stage procedure can then be used for standard instrumental variable analysis. Though it is beyond the scope of this article to describe exactly how to use these models for IV analysis, if the second stage is linear, typically the regression coefficient would be used to determine the significance of the treatment variable on the outcome. If the second stage is nonlinear, other types of statistical inference techniques can be used, or variable importance techniques.

## 8. Conclusion and Discussion

As the current trend towards nonlinear modeling choices continues, particularly in instrumental variable analysis, we must always check for validity of instruments and misspecification of modeling choices, and fix misspecified models if possible. If a scientist proceeds with analysis and leaves validity and modeling choices as an afterthought, it is possible that their conclusions could be wrong. Our approach aims to prevent this.

The contributions of this paper are: (i) A framework that generalizes instrumental variable analysis to general nonlinear modeling. The framework can theoretically handle any choices for nonlinear models, and for instance, could be extended beyond the choices made in this paper to very complex model choices such as neural networks or combinations of decision trees. (ii) An empirical validity check for the presence of valid instruments or a misspecified model, or a lack of serendipity in the data or fitting procedure. This empirical validity check is incorporated as a constraint in the optimization problem solved in the first stage of the analysis. As we showed, it is often possible to fix a misspecified model (or lack of serendipity), but one cannot fix a non-instrument. (iii) A new one-stage method for instrumental variable analysis, which is more flexible than the two-stage method, but may be harder to use in practice because it requires the solution of a more challenging optimization problem. In our experiments we used tools such as tensorflow, cvxopt and cvxpi, and as these automatic solvers continue to be developed, the one-stage approach may become more feasible in the future. (iv) Closed form solutions for the new two-stage formulation for linear models and general additive models. (v) Conditions under which the new IV formulation yields either the same solution as the traditional approach, no solutions, or potentially better solutions.

Possible directions for future work include developing approximate solutions to the framework’s one-stage and two-stage optimization problems for different loss functions, and working out theory of existence of solutions for these loss functions as we have done for the linear and general additive two-stage cases. Another possible future direction is in applications: one can apply our method to deep learning or other nonlinear analysis choices, or in more traditional modeling cases, as desired.

## Figures and Tables

**Table 1:** Potential optimization results we might find and intuition of what they mean for practice.

Optimization Result	Intuition
The constraint is never active.	The validity check always holds, so it is not useful and we cannot check whether the instrument is valid or not.
There is no feasible solution.	The validity check is not satisfied. The selected variable is not a valid instrument, and we cannot fix it.
The constraint is active, and there is a feasible solution.	The validity check was not initially satisfied, which means the proposed instrument initially appeared to be invalid. But we have fixed the model to make it appear valid.

**Table 2:** Potential optimization results under different model constructions in the case of **uni-dimensional** instruments. LM stands for linear model and GAM stands for general additive model. Blue indicates cases where the constraints introduced in this paper are relevant (active).

Prediction Model in Stage One	Prediction Model in Stage Two	Potential Optimization Results
LM	LM	The constraint is always satisfied (i.e., never active). (Theorem 2)
LM	GAM	The constraint is always satisfied (i.e., never active). (Theorem 3)
GAM	LM	EITHER There is a feasible solution & constraint is not active OR There is no feasible solution (Theorem 1)
GAM	GAM	EITHER There is a feasible solution & constraint is not active OR There is no feasible solution OR There exists a feasible solution and the constraint is active

**Table 3:** Potential optimization results under different model constructions in the case of a **multi-dimensional** instrument. LM stands for linear model and GAM stands for general additive model. Blue indicates cases where the constraints introduced in this paper are relevant (active).

Prediction Model in Stage One	Prediction Model in Stage Two	Potential Optimization Results
LM	LM	EITHER There is a feasible solution, constraint is not active OR There is no feasible solution (Theorem 1)
LM	GAM	EITHER There is a feasible solution, constraint is not active OR There is no feasible solution OR There is a feasible solution and the constraint is active
GAM	LM	EITHER There is a feasible solution, constraint is not active OR There is no feasible solution (Theorem 1)
GAM	GAM	EITHER There is a feasible solution, constraint is not active OR There is no feasible solution OR There is a feasible solution and the constraint is active

**Table 4:** Confusion Matrix by Our Two Stage Method (*γ* = 1%)

n = 2000	Predicted to be invalid. (There is no feasible solution.)	Passes validity check. (There is a feasible solution.)
Not an instrument	1000	0
Valid instrument	0	1000

**Table 5:** Confusion Matrix by Our Two Stage Method (*γ* = 0.1%)

n = 2000	Predicted to be invalid. (There is no feasible solution.)	Predicted to be valid. (There is a feasible solution.)
Not an instrument	797	203
Valid instrument	38	962

**Table 6:** Values of loss on both sides of the constraint

	*loss*(*r*, 0)	*loss*(*r*, $\hat{r}$)	True percentage: *loss*(*r*, $\hat{r}$)/*loss*(*r*, 0)
Linear first stage (insufficient): *X_t_* = (*x*, *z*)	44.44	34.50	77.64 (insufficient)%
Quadratic first stage (sufficient): *X_t_* = (*x*, *z*, *z*^2^)	34.51	34.50	99.98 (sufficient)%

**Table 7:** Values of loss on both sides on the constraint

	*loss*(*r*, 0)	*loss*(*r*, $\hat{r}$)	True percentage: *loss*(*r*, $\hat{r}$)/*loss*(*r*, 0)
Quadratic first stage: *X_t_* = (*x*, *z*, *z*^2^)	42.9038	35.4535	82.63%
Cubic first stage: *X_t_* = (*x*, *z*, *z*^2^, *z*^3^)	42.9131	35.3490	82.64%

**Table 8:** Comparison of four different models (from the simplest to the most complicated)

Models (Input Features)	RMSE of Outcomes *y*	Causal Effect	Neyman Variance
Model 1 Linear Regression (with only linear features)	0.7075	−0.4541	2.6139×10^−2^
Model 2 Logistic Regression (with only linear features)	0.7059	−0.4740	2.6017 ×10^−2^
Model 3 Logistic Regression (adding non-linear features of the covariates *x*)	0.6956	−0.4742	2.7239× 10^−2^
Model 4 Logistic Regression (adding interaction terms between the instrument *z* and the covariates *x*, the predicted values of the treatment $\hat{t}$ and the covariates *x*)	0.6731	−0.4992	2.9348×10^−2^

## Appendix A. Pseudocode for the Two-Stage and One-Stage ML-IV Methods

In order to generalize the square loss, we replace the inner product in the square loss function with a kernel function, which generalizes the least square solution to the kernel least square solution. The pseudo codes of the kernel least square methods are provided in this section. There are closed form solutions for these procedures that are analogous to the least square solutions, replacing the inner products with kernel functions. For cases beyond what we present, when there is not a closed form for the optimization problem, different techniques would be applied in practice to reach a local optimal solution. To solve the optimization problem in practice, we use solvers cvxopt and cvxpy in python for our two stage method and tensorflow for our one stage method.

### Two Stage Method

#### Stage One

1. construct the predictor matrix of *t* in Stage One, *X*^(*t*)^, where matrix *X*^(*t*)^ is defined element-wise by $x_{ij}^{\left(t\right)}\;=\;function_{j}(x_{i}.,z_{i})$, where *function* is a user-defined function, which creates features from *x_i_*. and *z_i._*;
2. compute $r_{1}^{*}$ by minimizing the square loss: $r_{1}^{*}\;\in \;{\text{argmin}}_{r_{1}}loss(r_{1})\;=\;\|t-X^{\left(t\right)}\omega_{r_{1}}{\|}_{2}^{2}$, where $\omega_{r_{1}}\;=\;X^{(t{)}^{T}}r_{1}=\sum_{i=1}^{n}x_{\;\;\;i\cdot }^{(t{)}^{T}}r_{1i}$, and obtain the least square solution ${\hat{\omega }}_{r_{1}}$, ${\hat{\omega }}_{r_{1}}\;=\;\sum_{i=1}^{n}{x^{\left(t\right)}}_{i\cdot }^{T}r_{1i}^{*}$;
3. obtain the least square solution of the treatment $\hat{t}$, ${\hat{t}}_{i\cdot }=x_{\;\;i\cdot }^{\left(t\right)}{\hat{\omega }}_{r_{1}}=x_{\;\;i\cdot }^{\left(t\right)}\sum_{i^{\prime }=1}^{n}x_{\;\;i^{\prime }\cdot }^{(t{)}^{T}}r_{1i^{\prime }}^{*}\;=\;\sum_{i^{\prime }=1}^{n}x_{\;\;i\cdot }^{\left(t\right)}x_{\;\;\;i^{\prime }\cdot }^{(t{)}^{T}}r_{1i^{\prime }}^{*}$;
4. if desired, replace the inner product with a kernel function *k*(·, ·), and obtain the kernel least square solution of $\hat{t}$, ${\hat{t}}_{i\cdot }\;=\;\sum_{i^{\prime }=1}^{n}k(x_{\;\;\;i\cdot }^{\left(t\right)},x_{\;\;\;i^{\prime }\cdot }^{\left(t\right)})r_{1i^{\prime }}^{*}$.

#### Stage Two

1. construct the predictor matrix of *y* in Stage One, *X*^(*y*)^, where matrix *X*^(*y*)^ is defined element-wise by $x_{ij}^{\left(y\right)}\;=\;function_{j}(x_{i\cdot },{\hat{t}}_{i})$, where *function* is a user-defined function, which creates features from *x_i._* and ${\hat{t}}_{i}$;
2. compute $r_{2}^{*}$ by minimizing the square loss:
  $$r_{2}^{*}\in {\text{argmin}}_{r_{2}}loss(r_{2})=\|y-X^{\left(y\right)}\beta_{r_{2}}{\|}_{2}^{2}+C\|H\vec{y}-HX^{\left(y\right)}\beta_{r_{2}}{\|}_{2}^{2},$$
  where $H\;=\;X_{t}(X_{t}^{T}X_{t}{)}^{-1}X_{t}^{T}$ and $\beta_{r_{2}}\;=\;X^{(y{)}^{T}}r_{2}=\sum_{i=1}^{n}x_{\;\;\;i\cdot }^{(t{)}^{T}}r_{2i}$, and obtain the least square solution of ${\hat{\beta }}_{r_{2}}$, ${\hat{\beta }}_{r_{2}}\;=\;\sum_{i=1}^{n}x_{\;\;\;i^{\prime }\cdot }^{(y{)}^{T}}r_{2i}^{*}$;
3. obtain the least square solution of the outcomes $\hat{y}$, ${\hat{y}}_{i\cdot }=x_{\;\;\;i\cdot }^{\left(y\right)}{\hat{\beta }}_{r_{2}}=x_{\;\;\;i\cdot }^{\left(y\right)}\sum_{i^{\prime }=1}^{n}x_{\;\;\;i^{\prime }\cdot }^{(y{)}^{T}}r_{2i^{\prime }}^{*}=\sum_{i^{\prime }=1}^{n}x_{\;\;\;i\cdot }^{\left(y\right)}x_{\;\;\;i^{\prime }\cdot }^{(y{)}^{T}}r_{2i^{\prime }}^{*}$;
4. if desired, replace the inner product with a kernel function *k*(·, ·), and obtain the kernel least square solution of $\hat{y}$, ${\hat{y}}_{i\cdot }\;=\;\sum_{i^{\prime }=1}^{n}k(x_{\;\;\;i\cdot }^{\left(y\right)},x_{\;\;\;i^{\prime }\cdot }^{\left(y\right)})r_{2i^{\prime }}^{*}$.

### One Stage Method

1. construct the predictor matrix of *t* in Stage One, *X*^(*t*)^, where matrix *X*^(*t*)^ is defined element-wise by $x_{ij}^{\left(t\right)}\;=\;function_{j}(x_{i\cdot },z_{i\cdot })$ and the predictor matrix of *y* in Stage One, *X*^(*y*)^, where matrix *X*^(*y*)^ is defined element-wise by $x_{ij}^{\left(y\right)}\;=\;function_{j}(x_{i\cdot },{\hat{t}}_{i})$;
2. compute $r_{1}^{*}$ and $r_{2}^{*}$ by minimizing the square loss:
  $$loss(r_{1},r_{2})=\|y-X^{\left(y\right)}(r_{1})\beta_{r_{2}}{\|}^{2}+C_{1}\|t-X^{\left(t\right)}\omega_{r_{1}}{\|}^{2}+C_{2}\|H\vec{y}-HX^{\left(y\right)}(r_{1})\beta_{r_{2}}{\|}^{2},$$
  where $H\;=\;X_{t}(X_{t}^{T}X_{t}{)}^{-1}X_{t}^{T}$, and where $X^{\left(y\right)}(r_{1})\;=\;function(\vec{X},\hat{t}(r_{1}))$, with $\hat{t}(r_{1})\;=\;X^{(t{)}^{T}}\omega_{r_{1}}$, where $\omega_{r_{1}}\;=\;X^{(t{)}^{T}}r_{1}=\sum_{i=1}^{n}x_{\;\;\;i\cdot }^{(t{)}^{T}}r_{1i}$, and also $\beta_{r_{2}}\;=\;X^{(y{)}^{T}}r_{2}=\sum_{i=1}^{n}x_{\;\;\;i\cdot }^{(y{)}^{T}}r_{2i}$. We then obtain the least square solution of ${\hat{\omega }}_{r_{1}}$ as ${\hat{\omega }}_{r_{1}}\;=\;\sum_{i=1}^{n}x_{\;\;\;i\cdot }^{(t{)}^{T}}r_{1i}^{*}$ and the least squares solution of ${\hat{\beta }}_{r_{2}}$ as ${\hat{\beta }}_{r_{2}}\;=\;\sum_{i=1}^{n}x_{\;\;\;i\cdot }^{(y{)}^{T}}r_{2i}^{*}$;
3. obtain the least square solutions of the treatment $\hat{t}$, ${\hat{t}}_{i\cdot }\;=\;x_{\;\;\;i\cdot }^{\left(t\right)}{\hat{\omega }}_{r_{1}}\;=\;x_{\;\;\;i\cdot }^{\left(t\right)}\sum_{i^{\prime }=1}^{n}x_{\;\;\;i^{\prime }\cdot }^{(t{)}^{T}}r_{1i^{\prime }}^{*}\;=\;\sum_{i^{\prime }=1}^{n}x_{\;\;\;i\cdot }^{\left(t\right)}x_{\;\;\;i^{\prime }\cdot }^{(t{)}^{T}}r_{1i^{\prime }}^{*}$ and the outcomes $\hat{y}$, ${\hat{y}}_{i\cdot }\;=\;x_{\;\;\;i\cdot }^{\left(y\right)}{\hat{\beta }}_{r_{2}}\;=\;x_{\;\;\;i\cdot }^{\left(y\right)}\sum_{i^{\prime }=1}^{n}x_{\;\;\;i^{\prime }\cdot }^{(y{)}^{T}}r_{2i^{\prime }}^{*}\;=\;\sum_{i^{\prime }=1}^{n}x_{\;\;\;i\cdot }^{\left(y\right)}x_{\;\;\;i^{\prime }\cdot }^{(y{)}^{T}}r_{2i^{\prime }}^{*}$;
4. if desired, replace the inner product with a kernel function *k*(·, ·), and obtain the kernel least square solutions of $\hat{t}$, ${\hat{t}}_{i\cdot }\;=\;\sum_{i^{\prime }=1}^{n}k(x_{\;\;\;i\cdot }^{\left(t\right)},x_{\;\;\;i^{\prime }\cdot }^{\left(t\right)})r_{1i^{\prime }}^{*}$ and $\hat{y}$, ${\hat{y}}_{i\cdot }\;=\;\sum_{i^{\prime }=1}^{n}k(x_{\;\;\;i\cdot }^{\left(y\right)},x_{\;\;\;i^{\prime }\cdot }^{\left(y\right)})r_{2i^{\prime }}^{*}$.

## Appendix B. Proofs

**Lemma 1** *If the models in Stage One and Stage Two are both linear models, the optimal (minimum) solution of the objective function (2) (i.e.,* ${\hat{\beta }}_{\text{min}}\;=\;\text{arg}\;{\text{min}}_{\beta }\;\beta^{T}X_{y}^{T}X_{y}\beta -2\beta^{T}X_{y}^{T}\vec{y}+{\vec{y}}^{T}\vec{y}$*) equals the optimal (minimum) solution of the objective function on the left side of Constraint (3) (i.e.,* ${\hat{\beta }}_{\text{min}}^{\prime }\;=\;\text{arg}\;{\text{min}}_{\beta }\;\beta^{T}X_{y}^{T}HX_{y}\beta -2\beta^{T}X_{y}^{T}H\vec{y}+{\vec{y}}^{T}H\vec{y}$*), i.e.,* ${\hat{\beta }}_{\text{min}}={\hat{\beta }}_{\text{min}}^{\prime }$. *This statement is true regardless of the dimension of* $\vec{X}$ *and the dimension of* $\vec{Z}$.

**Proof.** Recall the notation for the model in Stage One is: $\hat{t}\;=\;f_{\omega }(\vec{X},\vec{Z})\;=\;f_{\omega }(x_{1},\cdots ,x_{p},z_{1},\cdots ,z_{q})$. To notate that it is a linear model, we use the following notation: $\hat{t}=\omega_{1}x_{1}+\cdots +\omega_{p}x_{p}+\omega_{p+1}z_{1}+\cdots +\omega_{p+q}z_{q}$.

Recall notation for the model in Stage Two is: $\hat{y}\;=\;g_{\beta }(\vec{X},\hat{t})\;=\;g_{\beta }(x_{1},\cdots ,x_{p},\hat{t})$. To notate that it is a linear model, we use the following notation: $\hat{y}\;=\;\beta_{1}x_{1}+\cdots +\beta_{p}x_{p}+\beta_{p+1}\hat{t}$.

Define the predictor matrix of *t* in Stage One:

$$X_{t}=(x_{1},\cdots ,x_{p},z_{1},\cdots ,z_{q}{)}_{n\times (p+q)}\in R^{n\times (p+q)}.$$

Define the predictor matrix of *y* in Stage Two:

$$X_{y}=(x_{1},\cdots ,x_{p},\hat{t}{)}_{n\times (p+1)}\;\;=(x_{1},\cdots ,x_{p},z_{1},\cdots ,z_{q}{)}_{n\times (p+q)}{\left(\begin{array}{cccc} 1 & \cdots & 0 & \omega_{1}\\ \vdots & & \vdots & \vdots \\ 0 & \cdots & 1 & \omega_{p}\\ 0 & \cdots & 0 & \omega_{p+1}\\ \vdots & & \vdots & \vdots \\ 0 & \cdots & 0 & \omega_{p+q} \end{array}\right)}_{\left(p+q)\times (p+1\right)}\;\;≕X_{t}B\in R^{n\times (p+1)}.$$

In Stage Two of our two stage method, the optimal (minimum) solution of the objective function (2) ($\beta^{T}X_{y}^{T}X_{y}\beta -2\beta^{T}X_{y}^{T}\vec{y}+{\vec{y}}^{T}\vec{y}$) is ${\hat{\beta }}_{\text{min}}\;=\;[X_{y}^{T}X_{y}{]}^{-1}X_{y}^{T}\vec{y}$, and the optimal (minimum) solution of the objective function of Constraint (3) ($\left(\beta^{T}X_{y}^{T}HX_{y}\beta -2\beta^{T}X_{y}^{T}H\vec{y}+{\vec{y}}^{T}H\vec{y}\right)$) is ${\hat{\beta }}_{\text{min}}^{\prime }\;=\;[X_{y}^{T}HX_{y}{]}^{-1}X_{y}^{T}H\vec{y}$. Then,

$${\hat{\beta }}_{\text{min}}^{\prime }=[X_{y}^{T}HX_{y}{]}^{-1}X_{y}^{T}H\vec{y}\;\;=[B^{T}X_{t}^{T}HX_{t}B{]}^{-1}B^{T}X_{t}^{T}H\vec{y},\;\text{where}\;X_{y}=X_{t}B\;\;=[B^{T}X_{t}^{T}X_{t}[X_{t}^{T}X_{t}{]}^{-1}X_{t}^{T}X_{t}B{]}^{-1}B^{T}X_{t}^{T}X_{t}[X_{t}^{T}X_{t}{]}^{-1}X_{t}^{T}\vec{y},\;\text{where}\;X_{y}=X_{t}B\;\;=[B^{T}X_{t}^{T}X_{t}B{]}^{-1}B^{T}X_{t}^{T}\vec{y}\;\;={\hat{\beta }}_{\text{min}}.$$

Therefore, the two optimal solutions are equivalent, i.e., ${\hat{\beta }}_{\text{min}}={\hat{\beta }}_{\text{min}}^{\prime }$. ■

**Lemma 2** *If the model in Stage One is a general additive model, and the model in Stage Two is a linear model, then the result of Lemma 1 still holds. This statement is true regardless of the dimension of* $\vec{X}$ and the dimension of $\vec{Z}$.

**Proof**. Recall the notation for the model in Stage One is: $\hat{t}\;=\;f_{\omega }(\vec{X},\vec{Z})\;=\;f_{\omega }(x_{1},\cdots ,x_{p},z_{1},\cdots ,z_{q})$. To notate that it is a general additive model, we use the following notation: $\hat{t}=\omega_{1}x_{1}+\cdots +\omega_{p}x_{p}+\omega_{p+1}z_{1}+\cdots +\omega_{p+q}z_{q}+\omega_{p+q+1}{\text{feature}}_{1}(\vec{X},\vec{Z})+\cdots +\omega_{p+q+k}{\text{feature}}_{k}(\vec{X},\vec{Z})$, where ${\text{feature}}_{j}(\vec{X},\vec{Z})$, *j* = 1, ⋯ , *k* is a non-linear function of ($\vec{X}$, $\vec{Z}$).

Recall notation for the model in Stage Two is: $\hat{y}\;=\;g_{\beta }(\vec{X},\hat{t})\;=\;g_{\beta }(x_{1},\cdots ,x_{p},\hat{t})$. To notate that it is a linear model, we use the following notation: $\hat{y}\;=\;\beta_{1}x_{1}+\cdots +\beta_{p}x_{p}+\beta_{p+1}\hat{t}$.

Define the predictor matrix of *t* in Stage One:

$$X_{t}=(x_{1},\cdots ,x_{p},z_{1},\cdots ,z_{q},{\text{feature}}_{1}(\vec{X},\vec{Z}),\cdots ,{\text{feature}}_{k}(\vec{X},\vec{Z}){)}_{n\times (p+q+k)}\in R^{n\times (p+q+k)}.$$

Define the predictor matrix of *y* in Stage Two:

$$X_{y}=(x_{1},\cdots ,x_{p},\hat{t}{)}_{n\times (p+1)}\;\;=(x_{1},\cdots ,x_{p},z_{1},\cdots ,z_{q},{\text{feature}}_{1}(\vec{X},\vec{Z}),\cdots ,{\text{feature}}_{k}(\vec{X},\vec{Z}){)}_{n\times (p+q+k)}\;\;\cdot {\left(\begin{array}{cccc} 1 & \cdots & 0 & \omega_{1}\\ \vdots & & \vdots & \vdots \\ 0 & \cdots & 1 & \omega_{p}\\ 0 & \cdots & 0 & \omega_{p+1}\\ \vdots & & \vdots & \vdots \\ 0 & \cdots & 0 & \omega_{p+q}\\ 0 & \cdots & 0 & \omega_{p+q+1}\\ \vdots & & \vdots & \vdots \\ 0 & \cdots & 0 & \omega_{p+q+k} \end{array}\right)}_{\left(p+q+k)\times (p+1\right)}\;\;≕X_{t}B^{\prime }\in R^{n\times (p+1)}.$$

Since the matrix *B*′ has the same form as the matrix *B* in Lemma 1, it has already been proved that two optimal (minimum) solutions are equivalent i.e. ${\hat{\beta }}_{\text{min}}\;=\;{\hat{\beta }}_{\text{min}}^{\prime }$. ■

**Lemma 3** *If the models in Stage One and Stage two are general additive models that have the following forms:*

$$\hat{t}=\omega_{1}{\text{feature}}_{1}(\vec{X})+\cdots +\omega_{k_{1}}{\text{feature}}_{k_{1}}(\vec{X})+\omega_{k_{1}+1}{\text{feature}}_{k_{1}+1}(\vec{X},\vec{Z})+\cdots +\;\;\omega_{k_{1}+k_{2}}{\text{feature}}_{k_{1}+k_{2}}(\vec{X},\vec{Z})$$

*where* ${\text{feature}}_{j}(\vec{X})$, *j* = 1, ⋯ , *k*_1_ *is a linear or non-linear function of* $\vec{X}$ *and* ${\text{feature}}_{j}(\vec{X},\vec{Z})$, *j* = *k*_1_ + 1, ⋯ , *k*_1_ + *k*_2_ *is a linear or non-linear function of* ($\vec{X}$, $\vec{Z}$) *or only* $\vec{Z}$*, and*

$$\hat{y}=\beta_{1}{\text{feature}}_{1}(\vec{X})+\cdots +\beta_{k_{1}^{\prime }}{\text{feature}}_{k_{1}^{\prime }}(\vec{X})+\beta_{k_{1}^{\prime }+1}\hat{t}$$

*where* $k_{1}^{\prime }\;\leq \;k_{1}$ *and* $\{{\text{feature}}_{j}(\vec{X}){\}}_{j=1}^{k_{1}^{\prime }}$ *is a subset of* $\{{\text{feature}}_{j}(\vec{X}){\}}_{j=1}^{k_{1}}$*, then the result of Lemma 1 still holds. In other words, if the following conditions are satisfied:*

1. *the input features of the covariates* $\vec{X}$ *in Stage Two are a subset of those in Stage One;*
2. *the model in Stage Two contains only the linear term of the predicted values of the treatment* $\hat{t}$,

*then the result of Lemma 1 still holds. This statement is true regardless of the dimension of* $\vec{X}$ *and the dimension of* $\vec{Z}$. *Note that Lemma 3 is a more general version of both Lemma 1 and Lemma 2.*

**Proof**. Recall the notation of the models in Stage One and Stage Two in Lemma 3 and define the predictor matrices as follows:

Define the predictor matrix of *t* in Stage One:

$$X_{t}=({\text{feature}}_{1}(\vec{X}),\cdots ,{\text{feature}}_{k_{1}}(\vec{X}),{\text{feature}}_{k_{1}+1}(\vec{X},\vec{Z}),\cdots ,{\text{feature}}_{k_{1}+k_{2}}(\vec{X},\vec{Z}){)}_{n\times (k_{1}+k_{2})}\;\;\in R^{n\times (k_{1}+k_{2})}.$$

Define the predictor matrix of *y* in Stage Two:

$$X_{y}=({\text{feature}}_{1}(\vec{X}),\cdots ,{\text{feature}}_{k_{1}^{\prime }}(\vec{X}),\hat{t}{)}_{n\times (p+1)}\;\;=({\text{feature}}_{1}(\vec{X}),\cdots ,{\text{feature}}_{k_{1}}(\vec{X}),{\text{feature}}_{k_{1}+1}(\vec{X},\vec{Z}),\cdots ,{\text{feature}}_{k_{1}+k_{2}}(\vec{X},\vec{Z}){)}_{n\times (k_{1}+k_{2})}\;\;\cdot {\left(\begin{array}{cccc} 1 & \cdots & 0 & \omega_{1}\\ \vdots & & \vdots & \vdots \\ 0 & \cdots & 1 & \omega_{k_{1}^{\prime }}\\ 0 & \cdots & 0 & \omega_{k_{1}^{\prime }+1}\\ \vdots & & \vdots & \vdots \\ 0 & \cdots & 0 & \omega_{k_{1}}\\ 0 & \cdots & 0 & \omega_{k_{1}+1}\\ \vdots & & \vdots & \vdots \\ 0 & \cdots & 0 & \omega_{k_{1}+k_{2}} \end{array}\right)}_{\left(k_{1}+k_{2})\times (k_{1}^{\prime }+1\right)}\;\;≕X_{t}B^{\prime \prime }\in R^{n\times (k_{1}^{\prime }+1)}.$$

Since the matrix *B*″ has the same form as the matrix *B* in Lemma 1, it has already been proved that two optimal (minimum) solutions are equivalent i.e. ${\hat{\beta }}_{\text{min}}\;=\;{\hat{\beta }}_{\text{min}}^{\prime }$. ■

**Theorem 1 (Partial)** *If the model in Stage Two is a linear model, and if the model in Stage One is a linear model or a general additive model, either Constraint (3) (*$\beta^{T}X_{y}^{T}HX_{y}\beta -2\beta^{T}X_{y}^{T}H\vec{y}+{\vec{y}}^{T}H\vec{y}\leq \epsilon^{\prime }$*) is not active or there is no feasible solution. This statement is true regardless of the dimension of* $\vec{X}$ *and the dimension of* $\vec{Z}$.

**Proof**. To notate the objective function 2 ($\beta^{T}X_{y}^{T}HX_{y}\beta -2\beta^{T}X_{y}^{T}H\vec{y}+{\vec{y}}^{T}H\vec{y}$), we use the following notation: $C_{\beta^{\prime }}(x_{1},\cdots ,x_{p},\hat{t})$.

To notate the objective function on the left side of Constraint 3 ($\beta^{T}X_{y}^{T}HX_{y}\beta -2\beta^{T}X_{y}^{T}H\vec{y}+{\vec{y}}^{T}H\vec{y}$), we use the following notation: $C_{\beta^{\prime }}(x_{1},\cdots ,x_{p},\hat{t})$.

Recall Lemma 1 and Lemma 2, the optimal (minimum) solution of the objective function 2 ($C_{\beta }(x_{1},\cdots ,x_{p},\hat{t})$) is ${\hat{\beta }}_{\text{min}}$, and the optimal (minimum) solution of the objective function on the left side of Constraint 3 ($C_{\beta^{\prime }}(x_{1},\cdots ,x_{p},\hat{t})$) is ${\hat{\beta }}_{\text{min}}^{\prime }$. Two optimal (minimum) solutions are equivalent, i.e., ${\hat{\beta }}_{\text{min}}={\hat{\beta }}_{\text{min}}^{\prime }$.

If Constraint 3 is active, then ${\hat{\beta }}_{\text{min}}$ does not satisfy it, i.e., $C_{{\hat{\beta }}_{\text{min}}}(x_{1},\cdots ,x_{p},\hat{t})>\epsilon^{\prime }$.

$$\underset{\beta^{\prime }}{\text{min}}\;C_{\beta^{\prime }}(x_{1},\cdots ,x_{p},\hat{t})\;\;=\;C_{{\hat{\beta }}_{\text{min}}^{\prime }}(x_{1},\cdots ,x_{p},\hat{t}),\;\text{where}\;{\hat{\beta }}_{\text{min}}^{\prime }\;\text{is the optimal (minimum) solution of}\;C_{\beta^{\prime }}(x_{1},\cdots ,x_{p},\hat{t})\;\;=\;C_{{\hat{\beta }}_{\text{min}}}(x_{1},\cdots ,x_{p},\hat{t})>\epsilon^{\prime },\;\text{where}\;{\hat{\beta }}_{\text{min}}^{\prime }={\hat{\beta }}_{\text{min}}.$$

Therefore, $C_{\beta^{\prime }}(x_{1},\cdots ,x_{p},\hat{t})>\epsilon^{\prime }$, Constraint 3 is never satisfied. In conclusion, once Constraint 3 ($\beta^{T}X_{y}^{T}HX_{y}\beta -2\beta^{T}X_{y}^{T}H\vec{y}+{\vec{y}}^{T}H\vec{y}\leq \epsilon^{\prime }$) is active, there does not exist a solution that can satisfy it. ■

**Theorem 1 (Full)** *If the models in Stage One and Stage two are general additive models that have the following forms:*

$$\hat{t}=\omega_{1}{\text{feature}}_{1}(\vec{X})+\cdots +\omega_{k_{1}}{\text{feature}}_{k_{1}}(\vec{X})+\omega_{k_{1}+1}{\text{feature}}_{k_{1}+1}(\vec{X},\vec{Z})+\cdots +\;\;\omega_{k_{1}+k_{2}}{\text{feature}}_{k_{1}+k_{2}}(\vec{X},\vec{Z})$$

*where each* ${\text{feature}}_{j}(\vec{X})$, *j* = 1, ⋯ , *k*_1_ *is a linear or non-linear function of* $\vec{X}$ *and each* ${\text{feature}}_{j}(\vec{X},\vec{Z})$, *j* = *k*_1_ + 1, ⋯ , *k*_1_ + *k*_2_ *is a linear or non-linear function of* ($\vec{X}$, $\vec{Z}$) *or only* $\vec{Z}$*, and*

$$\hat{y}=\beta_{1}{\text{feature}}_{1}(\vec{X})+\cdots +\beta_{k_{1}^{\prime }}{\text{feature}}_{k_{1}^{\prime }}(\vec{X})+\beta_{k_{1}^{\prime }+1}\hat{t}$$

*where* $k_{1}^{\prime }\leq k_{1}$ *and* $\{{\text{feature}}_{j}(\vec{X}){\}}_{j=1}^{k_{1}^{\prime }}$ *is a subset of* $\{{\text{feature}}_{j}(\vec{X}){\}}_{j=1}^{k_{1}}$*, then either Constraint 3 (*$\beta^{T}X_{y}^{T}HX_{y}\beta -2\beta^{T}X_{y}^{T}H\vec{y}+{\vec{y}}^{T}H\vec{y}\leq \epsilon^{\prime }$*) is not active or there is no feasible solution. This statement is true regardless of the dimension of* $\vec{X}$ *and the dimension of* $\vec{Z}$*. In other words, if the following conditions are satisfied:*

1. the input features of the covariates $\vec{X}$ in Stage Two is a subset of those in Stage One;
2. the model in Stage Two only contains the linear term of the predicted values of the treatment $\hat{t}$,

*then either Constraint* (3) *(i.e.,* $\beta^{T}X_{y}^{T}HX_{y}\beta -2\beta^{T}X_{y}^{T}H\vec{y}+{\vec{y}}^{T}H\vec{y}\leq \epsilon^{\prime }$*) is not active or there is no feasible solution*.

**Proof**. The proof of the Full Version of Theorem 1 using Lemma 3 is the same as the proof of Theorem 1 using Lemma 1 and Lemma 2. ■

**Theorem 2 (Partial)** *In the uni-dimensional case of the instrument* $\vec{Z}$*, if the models in Stage One and Stage Two are both linear models, Constraint* (3) *(*$\beta^{T}X_{y}^{T}HX_{y}\beta -2\beta^{T}X_{y}^{T}H\vec{y}+{\vec{y}}^{T}H\vec{y}\leq \epsilon^{\prime }$*) is always satisfied (i.e., never active). This statement is true regardless of the dimension of* $\vec{X}$.

**Proof**. Recall the notation for the model in Stage One is: $\hat{t}=f_{\omega }(\vec{X},\vec{Z})\;=\;f_{\omega }(x_{1},\cdots ,x_{p},z_{1})$. To notate that it is a linear model, we use the following notation: $\hat{t}=\omega_{1}x_{1}+\cdots +\omega_{p}x_{p}+\omega_{p+1}z_{1}$.

Recall notation for the model in Stage Two is: $\hat{y}\;=\;g_{\beta }(\vec{X},\hat{t})\;=\;g_{\beta }(x_{1},\cdots ,x_{p},\hat{t})$. To denote that it is a linear model, we use the following notation: $\hat{y}=\beta_{1}x_{1}+\cdots +\beta_{p}x_{p}+\beta_{p+1}\hat{t}$.

Define the predictor matrix of *t* in Stage One:

$$X_{t}=(x_{1},x_{2},\cdots ,x_{p},z_{1}{)}_{n\times (p+1)}\in R^{n\times (p+1)}$$

Define the predictor matrix of *y* in Stage Two:

$$X_{y}=(x_{1},x_{2}\cdots ,x_{p},\hat{t}{)}_{n\times (p+1)}\;\;=(x_{1},x_{2},\cdots ,x_{p},z_{1}{)}_{n\times (p+1)}{\left(\begin{array}{ccccc} 1 & 0 & \cdots & 0 & \omega_{1}\\ 0 & 1 & \cdots & 0 & \omega_{2}\\ \vdots & \vdots & & \vdots & \vdots \\ 0 & 0 & \cdots & 1 & \omega_{p}\\ 0 & 0 & \cdots & 0 & \omega_{p+1} \end{array}\right)}_{\left(p+1)\times (p+1\right)}\;\;≕X_{t}A\in R^{n\times (p+1)}$$

where diagonal entries of *A* are *a_ii_* = 1, 1 ≤ *i* ≤ *p* and *a*_(*p*+1)(*p*+1)_ = *ω*_*p*+1_. Therefore, the upper trapezoidal matrix *A* has non-zero diagonal entries.

As shown in the following, the hat matrix *H*_1_ equals the hat matrix *H*.

$$H_{1}=X_{y}[X_{y}^{T}X_{y}{]}^{-1}X_{y}^{T},\;\text{where}\;X_{y}=X_{t}A\;\;=X_{t}A[A^{T}X_{t}^{T}X_{t}A{]}^{-1}A^{T}X_{t}^{T},\;\text{where}\;A\in R^{\left(p+1)\times (p+1\right)}\;\;=X_{t}AA^{-1}[X_{t}^{T}X_{t}{]}^{-1}A^{-T}A^{T}X_{t}^{T}\;\;=X_{t}[X_{t}^{T}X_{t}{]}^{-1}X_{t}^{T}\;\;=H.$$

In Stage Two of our two stage method, the optimal (minimum) solution of the objective function 2 ($\beta^{T}X_{y}^{T}X_{y}\beta -2\beta^{T}X_{y}^{T}\vec{y}+{\vec{y}}^{T}\vec{y}$) is ${\hat{\beta }}_{\text{min}}\;=\;[X_{y}^{T}X_{y}{]}^{-1}X_{y}^{T}\vec{y}$ and $\hat{y}\;=\;X_{y}{\hat{\beta }}_{\text{min}}\;=\;X_{y}[X_{y}^{T}X_{y}{]}^{-1}X_{y}^{T}\vec{y}=H_{1}\vec{y}$.

The objective function on the left side of Constraint 3 ($\beta^{T}X_{y}^{T}HX_{y}\beta -2\beta^{T}X_{y}^{T}H\vec{y}+{\vec{y}}^{T}H\vec{y}$) is:

$$\|H(\vec{y}-\hat{y}){\|}^{2}=\|H\vec{y}-H\hat{y}{\|}^{2}\;\text{where}\;\hat{y}=H_{1}y\;\;=\|H\vec{y}-HH_{1}\vec{y}{\|}^{2}\;\text{where}\;H_{1}=H\;\;=\|H\vec{y}-H^{2}\vec{y}{\|}^{2}\;\text{where}\;H^{2}=H\;\;=\|H\vec{y}-H\vec{y}{\|}^{2}\;\;=0\leq \epsilon^{\prime }\;\text{where}\;\epsilon^{\prime }>0.$$

Therefore, Constraint (3) ($\beta^{T}X_{y}^{T}HX_{y}\beta -2\beta^{T}X_{y}^{T}H\vec{y}+{\vec{y}}^{T}H\vec{y}\leq \epsilon^{\prime }$) is always satisfied (i.e., never active). ■

**Theorem 2 (Full)** *In the uni-dimensional case of the instrument* $\vec{Z}$*, if the models in Stage One and Stage Two are general additive models that have the following forms:*

$$\hat{t}=\omega_{1}{\text{feature}}_{1}(\vec{X})+\cdots +\omega_{k}{\text{feature}}_{k}(\vec{X})+\omega_{k+1}{\text{feature}}_{k+1}(\vec{Z})$$

*where each* ${\text{feature}}_{j}(\vec{X})$, *j* = 1, ⋯ , *k* *is a linear or non-linear function of* $\vec{X}$ *and* ${\text{feature}}_{k+1}(\vec{Z})$ *is a linear or non-linear function of* $\vec{Z}$*, and*

$$\hat{y}=\beta_{1}{\text{feature}}_{1}(\vec{X})+\cdots +\beta_{k}{\text{feature}}_{k}(\vec{X})+\beta_{k+1}\hat{t}$$

*where each* ${\text{feature}}_{j}(\vec{X})$, *j* = 1, ⋯ , *k is a linear or non-linear function of* $\vec{X}$*, then Constraint 3 (*$\beta^{T}X_{y}^{T}HX_{y}\beta -2\beta^{T}X_{y}^{T}H\vec{y}+{\vec{y}}^{T}H\vec{y}\leq \epsilon^{\prime }$*) is always satisfied (i.e., never active)*.

This statement is true regardless of the dimension of $\vec{X}$. In other words, if the following conditions are satisfied:

1. the models in Stage One and Stage two share the same input features of the covariates $\vec{X}$;
2. the model in Stage One contains only one input feature of the instrument $\vec{Z}$, which can be either linear or non-linear;
3. the model in Stage One contains no interaction term of the covariates $\vec{X}$ and the instrument $\vec{Z}$;
4. the model in Stage Two only contains the linear term of the predicted values of the treatment $\hat{t}$,

*then Constraint* 3 *(*$\beta^{T}X_{y}^{T}HX_{y}\beta -2\beta^{T}X_{y}^{T}H\vec{y}+{\vec{y}}^{T}H\vec{y}\leq \epsilon^{\prime }$*) is always satisfied (i.e., never active)*.

**Proof**. Recall the notation of the models in Stage One and Stage Two in the Full Version of Theorem 2 and define the predictor matrices as follows:

Define the predictor matrix of *t* in Stage One:

$$X_{t}=({\text{feature}}_{1}(\vec{X}),\cdots ,{\text{feature}}_{k}(\vec{X}),{\text{feature}}_{k+1}(\vec{Z}){)}_{n\times (k+1)}\in R^{n\times (k+1)}$$

Define the predictor matrix of *y* in Stage Two:

$$X_{y}=({\text{feature}}_{1}(\vec{X}),\cdots ,{\text{feature}}_{k}(\vec{X}),\hat{t}{)}_{n\times (k+1)}\;\;=({\text{feature}}_{1}(\vec{X}),\cdots ,{\text{feature}}_{k}(\vec{X}),{\text{feature}}_{k+1}(\vec{Z}){)}_{n\times (k+1)}{\left(\begin{array}{cccc} 1 & \cdots & 0 & \omega_{1}\\ \vdots & & \vdots & \vdots \\ 0 & \cdots & 1 & \omega_{k}\\ 0 & \cdots & 0 & \omega_{k+1} \end{array}\right)}_{\left(k+1)\times (k+1\right)}\;\;≕X_{t}A\in R^{n\times (k+1)}$$

where diagonal entries of *A* are *a_ii_* = 1, 1 ≤ *i* ≤ *k* and *a*_(*k*+1)(*k*+1)_ = *ω*_*k*+1_. Therefore, the upper trapezoidal matrix *A* has non-zero diagonal entries.

As shown in the following, the hat matrix *H*_1_ equals the hat matrix *H*.

$$H_{1}=X_{y}[X_{y}^{T}X_{y}{]}^{-1}X_{y}^{T},\;\text{where}\;X_{y}=X_{t}A\;\;=X_{t}A[A^{T}X_{t}^{T}X_{t}A{]}^{-1}A^{T}X_{t}^{T},\;\text{where}\;A\in R^{\left(k+1)\times (k+1\right)}\;\;=X_{t}AA^{-1}[X_{t}^{T}X_{t}{]}^{-1}A^{-T}A^{T}X_{t}^{T}\;\;=X_{t}[X_{t}^{T}X_{t}{]}^{-1}X_{t}^{T}\;\;=H.$$

In Stage Two of our two stage method, the optimal (minimum) solution of the objective function 2 ($\beta^{T}X_{y}^{T}X_{y}\beta -2\beta^{T}X_{y}^{T}\vec{y}+{\vec{y}}^{T}\vec{y}$) is ${\hat{\beta }}_{\text{min}}\;=\;[X_{y}^{T}X_{y}{]}^{-1}X_{y}^{T}\vec{y}$ and $\hat{y}\;=\;X_{y}{\hat{\beta }}_{\text{min}}\;=\;X_{y}[X_{y}^{T}X_{y}{]}^{-1}X_{y}^{T}\vec{y}=H_{1}\vec{y}$.

The objective function on the left side of Constraint 3 ($\beta^{T}X_{y}^{T}HX_{y}\beta -2\beta^{T}X_{y}^{T}H\vec{y}+{\vec{y}}^{T}H\vec{y}$) is:

$$\|H(\vec{y}-\hat{y}){\|}^{2}=\|H\vec{y}-H\hat{y}{\|}^{2}\;\text{where}\;\hat{y}=H_{1}y\;\;=\|H\vec{y}-HH_{1}y{\|}^{2}\;\text{where}\;H_{1}=H\;\;=\|H\vec{y}-H^{2}y{\|}^{2}\;\text{where}\;H^{2}=H\;\;=\|H\vec{y}-H\vec{y}{\|}^{2}\;\;=0\leq \epsilon^{\prime }\;\text{where}\;\epsilon^{\prime }>0.$$

Therefore, Constraint 3 ($\beta^{T}X_{y}^{T}HX_{y}\beta -2\beta^{T}X_{y}^{T}H\vec{y}+{\vec{y}}^{T}H\vec{y}\leq \epsilon^{\prime }$) is always satisfied (i.e., never active). ■

**Lemma 4** *If* $A\in R^{n\times m}$, *n* > *m is an upper trapezoidal matrix with non-zero diagonal entries, the matrix A* [*A^T^ A*]^−1^ *A^T^ is a block matrix of the form* $A[A^{T}A{]}^{-1}A^{T}=\left(\begin{array}{cc} I_{m\times m} & 0_{m\times (n-m)}\\ 0_{(n-m)\times m} & 0_{\left(n-m)\times (n-m\right)} \end{array}\right)$, *I*_*m*×*m*_ *is an m-dimensional identity matrix*.

**Proof**. Denote $A=\left(\begin{array}{c} C_{m\times m}\\ 0_{(n-m)\times m} \end{array}\right)$, where $C\;\in \;R^{m\times m}$ is an upper triangular matrix with non-zero diagonal entries.

$$A[A^{T}A{]}^{-1}A^{T}=\left(\begin{array}{c} C_{m\times m}\\ 0_{(n-m)\times m} \end{array}\right)[\left(C_{m\times m}^{T}\;\;0_{(n-m)\times m}\right)\left(\begin{array}{c} C_{m\times m}\\ 0_{(n-m)\times m} \end{array}\right){]}^{-1}\left(C_{m\times m}^{T}\;\;0_{(n-m)\times m}\right)\;\;=\left(\begin{array}{c} C_{m\times m}\\ 0_{(n-m)\times m} \end{array}\right)[C_{m\times m}^{T}C_{m\times m}{]}^{-1}\left(C_{m\times m}^{T}\;\;0_{(n-m)\times m}\right)\;\;=\left(\begin{array}{c} C_{m\times m}\\ 0_{(n-m)\times m} \end{array}\right)C_{m\times m}^{-1}C_{m\times m}^{-T}\left(C_{m\times m}^{T}\;\;0_{(n-m)\times m}\right)\;\;=\left(\begin{array}{c} I_{m\times m}\\ 0_{(n-m)\times m} \end{array}\right)\left(I_{m\times m}\;\;0_{(n-m)\times m}\right)\;\;=\left(\begin{array}{cc} I_{m\times m} & 0_{m\times (n-m)}\\ 0_{(n-m)\times m} & 0_{\left(n-m)\times (n-m\right)} \end{array}\right).$$

**Lemma 5** *If* $B\in R^{n\times n}$ *is an upper triangular matrix with non-zero diagonal entries, the matrix B*[*B^T^ B*]^−1^ *B^T^* = *I*_*n*×*n*_ *is an n-dimensional identity matrix*.

Proof.

$$B[B^{T}B{]}^{-1}B^{T}=BB^{-1}B^{-T}B^{T}=I_{n\times n}I_{n\times n}=I_{n\times n}.$$

**Theorem 3 (Partial)** *In the uni-dimensional case for the instrument* $\vec{Z}$*, if the model in Stage One is a linear model, and the model in Stage Two is a general additive model, Constraint* 3 *(*$\beta^{T}X_{y}^{T}HX_{y}\beta -2\beta^{T}X_{y}^{T}H\vec{y}+{\vec{y}}^{T}H\vec{y}\leq \epsilon^{\prime }$*) is never active. This statement is true regardless of the dimension of* $\vec{X}$.

**Proof**. Recall that Theorem 2 holds because *H* = *H*_1_. In Theorem 3, although *H* ≠ *H*_1_, *H* – *HH*_1_ = 0.

In Stage Two of our method, the optimal (minimum) solution of the objective function (2) ($\beta^{T}X_{y}^{T}X_{y}\beta -2\beta^{T}X_{y}^{T}\vec{y}+{\vec{y}}^{T}\vec{y}$) is ${\hat{\beta }}_{\text{min}}\;=\;[X_{y}^{T}X_{y}{]}^{-1}X_{y}^{T}\vec{y}$ and $\hat{y}\;=\;X_{y}{\hat{\beta }}_{\text{min}}\;=\;X_{y}[X_{y}^{T}X_{y}{]}^{-1}X_{y}^{T}\vec{y}=H_{1}\vec{y}$.

The objective function on the left side of Constraint (3) ($\beta^{T}X_{y}^{T}HX_{y}\beta -2\beta^{T}X_{y}^{T}H\vec{y}+{\vec{y}}^{T}H\vec{y}$) is:

$$\|H(\vec{y}-\hat{y}){\|}^{2}=\|H\vec{y}-H\hat{y}){\|}^{2}\;\;=\|H\vec{y}-HH_{1}y){\|}^{2}\;\text{where}\;\hat{y}=H_{1}y\;\;=\|(H-HH_{1})y){\|}^{2}\;\text{where}\;H-HH_{1}=0\;\;=0\leq \epsilon^{\prime }\;\text{where}\;\epsilon^{\prime }>0.$$

Therefore, Constraint (3) ($\beta^{T}X_{y}^{T}HX_{y}\beta -2\beta^{T}X_{y}^{T}H\vec{y}+{\vec{y}}^{T}H\vec{y}\leq \epsilon^{\prime }$) is always satisfied (i.e., never active).

**Next prove** *H* – *HH*_1_ = 0.

Recall the notation for the model in Stage One is: $\hat{t}\;=\;f_{\omega }(\vec{X},\vec{Z})\;=\;f_{\omega }(x_{1},\cdots ,x_{p},z_{1})$. To notate that it is a linear model, we use the following notation: $\hat{t}\;=\;\omega_{1}x_{1}+\cdots +\omega_{p}x_{p}+\omega_{p+1}z_{1}$.

Recall the notation for the model in Stage Two is: $\hat{y}\;=\;g_{\beta }(\vec{X},\hat{t})\;=\;g_{\beta }(x_{1},\cdots ,x_{p},\hat{t})$. To notate that it is a general additive model, we use the following notation: $\hat{y}\;=\;\beta_{1}x_{1}+\cdots +\beta_{p}x_{p}+\beta_{p+1}\hat{t}+\beta_{p+2}{\text{feature}}_{1}(\vec{X},\hat{t})+\cdots +\beta_{p+k+1}{\text{feature}}_{k}(\vec{X},\hat{t})$, where ${\text{feature}}_{j}(\vec{X},\hat{t})$, *j* = 1, ⋯ , *k* is a non-linear function of ($\vec{X}$, $\hat{t}$).

Define the predictor matrix of *t* in Stage One:

$$X_{t}=(x_{1},\cdots ,x_{p},z_{1})\in R^{n\times (p+1)}.$$

Define the predictor matrix of *y* in Stage Two:

$$X_{y}=(x_{1},\cdots ,x_{p},\hat{t},{\text{feature}}_{1}(\vec{X},\hat{t}),\cdots ,{\text{feature}}_{k}(\vec{X},\hat{t}))\in R^{n\times m}$$

where *m* = *p* + *k* + 1.

Use the Gram-Schmidt algorithm to construct an orthogonal set of unit vectors.

Step 1: *u*_1_ = *x*_1_, $e_{1}=\frac{u_{1}}{|u_{1}|}$

Step 2: $u_{2}=x_{2}-\frac{x_{2}\cdot x_{1}}{|u_{1}{|}^{2}}u_{1}$, $e_{2}=\frac{u_{2}}{|u_{2}|}$

.

.

.

Step p+1: $u_{p+1}=\hat{t}-\frac{\hat{t}\cdot u_{1}}{|u_{1}{|}^{2}}u_{1}-\cdots -\frac{\hat{t}\cdot u_{p}}{|u_{p}{|}^{2}}u_{p},\;e_{p+1}=\frac{u_{p+1}}{|u_{p+1}|}$

Step p+2: $u_{p+2}={\text{feature}}_{1}(\vec{X},\hat{t})-\frac{{\text{feature}}_{1}(\vec{X},\hat{t})\cdot u_{1}}{|u_{1}{|}^{2}}u_{1}-\cdots -\frac{{\text{feature}}_{1}(\vec{X},\hat{t})\cdot u_{p+1}}{|u_{p+1}{|}^{2}}u_{p+1}$, $e_{p+2}=\frac{u_{p+2}}{|u_{p+2}|}$

.

.

.

Step *m*: $u_{m}={\text{feature}}_{k}(\vec{X},\hat{t})-\frac{{\text{feature}}_{k}(\vec{X},\hat{t})\cdot u_{1}}{|u_{1}{|}^{2}}u_{1}-\cdots -\frac{{\text{feature}}_{k}(\vec{X},\hat{t})\cdot u_{m-1}}{|u_{m-1}{|}^{2}}u_{m-1}$, $e_{m}=\frac{u_{m}}{|u_{m}|}$.

Therefore the predictor matrix and *t* and *y* can be written as follows:

$$X_{t}=(x_{1},\cdots ,x_{p},z_{1})\;\;=(u_{1},u_{2},\cdots ,u_{m}){\left(\begin{array}{cccc} 1 & a_{12} & \cdots & a_{1(p+1)}\\ 0 & 1 & \cdots & a_{2(p+1)}\\ \vdots & \vdots & & \vdots \\ 0 & 0 & \cdots & 1∕\omega_{p+1}\\ 0 & 0 & \cdots & 0\\ \vdots & \vdots & & \vdots \\ 0 & 0 & \cdots & 0 \end{array}\right)}_{m\times (p+1)}\;\;≕UA_{u}=(e_{1},e_{2},\cdots ,e_{m}){\left(\begin{array}{cccc} |u_{1}| & 0 & \cdots & 0\\ 0 & |u_{2}| & \cdots & 0\\ 0 & 0 & \cdots & 0\\ 0 & 0 & \cdots & |u_{m}| \end{array}\right)}_{m\times m}{\left(\begin{array}{cccc} 1 & a_{12} & \cdots & a_{1(p+1)}\\ 0 & 1 & \cdots & a_{2(p+1)}\\ \vdots & \vdots & & \vdots \\ 0 & 0 & \cdots & 1∕\omega_{p+1}\\ 0 & 0 & \cdots & 0\\ \vdots & \vdots & & \vdots \\ 0 & 0 & \cdots & 0 \end{array}\right)}_{m\times (p+1)}\;\;≕ED_{u}A_{u}\;\;≕EA,$$

where diagonal entries of *D_u_* are *d_ii_* = ∣*u_i_*∣ > 0, and diagonal entries of *A_u_* are *a_ii_* = 1, 1 ≤ *i* ≤ *p* and *a*_(*p*+1)(*p*+1)_ – 1/*ω*_*p*+1_. Therefore, the upper trapezoidal matrix *A* = *D_u_A_u_* has non-zero diagonal entries.

$$X_{y}=(x_{1},\cdots ,x_{p},\hat{t},{\text{feature}}_{1}(\vec{X},\hat{t}),\cdots ,{\text{feature}}_{k}(\vec{X},\hat{t}))\;\;=(u_{1},u_{2},\cdots ,u_{m}){\left(\begin{array}{cccc} 1 & b_{12} & \cdots & b_{1m}\\ 0 & 1 & \cdots & b_{2m}\\ \vdots & \vdots & & \vdots \\ 0 & 0 & \cdots & 1 \end{array}\right)}_{m\times m}\;\;≕UB_{u}=(e_{1},e_{2},\cdots ,e_{m}){\left(\begin{array}{cccc} |u_{1}| & 0 & \cdots & 0\\ 0 & |u_{2}| & \cdots & 0\\ 0 & 0 & \cdots & 0\\ 0 & 0 & \cdots & |u_{m}| \end{array}\right)}_{m\times m}{\left(\begin{array}{cccc} 1 & b_{12} & \cdots & b_{1m}\\ 0 & 1 & \cdots & b_{2m}\\ \vdots & \vdots & & \vdots \\ 0 & 0 & \cdots & 1 \end{array}\right)}_{m\times m}\;\;≕ED_{u}B_{u}\;\;≕EB,$$

where diagonal entries of *D_u_* are *d_ii_* = ∣*u_i_*∣ > 0, and diagonal entries of *B_u_* are *b_ii_* = 1, 1 ≤ *i* ≤ *m*. Therefore, the upper triangular matrix *B* = *D_u_B_u_* has non-zero diagonal entries.

The hat matrix *H* is

$$H=X_{t}[X_{t}^{T}X_{t}{]}^{-1}X_{t}^{T},\;\text{where}\;X_{t}=EA\;\;=EA[A^{T}E^{T}EA{]}^{-1}A^{T}E^{T},\;\text{where}\;E^{T}E=I_{m}\;\;=EA[A^{T}A{]}^{-1}A^{T}E^{T}\;\;=E\left(\begin{array}{cc} I_{p+1} & 0_{\left(p+1)\times (m-p-1\right)}\\ 0_{\left(m-p-1)\times (p+1\right)} & 0_{m-p-1} \end{array}\right)E^{T}.$$

The hat matrix *H*_1_ is

$$H_{1}=X_{y}[X_{y}^{T}X_{y}{]}^{-1}X_{y}^{T},\;\text{where}\;X_{y}=EB\;\;=EB[B^{T}E^{T}EB{]}^{-1}B^{T}E^{T},\;\text{where}\;E^{T}E=I_{m}\;\;=EB[B^{T}B{]}^{-1}B^{T}E^{T}\;\;=EI_{m}E^{T}.$$

Therefore,

$$HH_{1}=E\left(\begin{array}{cc} I_{p+1} & 0_{\left(p+1)\times (m-p-1\right)}\\ 0_{\left(m-p-1)\times (p+1\right)} & 0_{m-p-1} \end{array}\right)E^{T}EI_{m}E^{T},\;\text{where}\;E^{T}E=I_{m}\;\;=E\left(\begin{array}{cc} I_{p+1} & 0_{\left(p+1)\times (m-p-1\right)}\\ 0_{\left(m-p-1)\times (p+1\right)} & 0_{m-p-1} \end{array}\right)I_{m}E^{T}\;\;=E\left(\begin{array}{cc} I_{p+1} & 0_{\left(p+1)\times (m-p-1\right)}\\ 0_{\left(m-p-1)\times (p+1\right)} & 0_{m-p-1} \end{array}\right)E^{T}\;\;=H.$$

**Theorem 3 (Full)** *In the uni-dimensional case for the instrument* $\vec{Z}$*, if models in Stage One and Stage Two are general additive models that have the following forms:*

$$\hat{t}=\omega_{1}{\text{feature}}_{1}(\vec{X})+\cdots +\omega_{k_{1}}{\text{feature}}_{k_{1}}(\vec{X})+\omega_{k_{1}+1}{\text{feature}}_{k_{1}+1}(\vec{Z})$$

*where each* ${\text{feature}}_{j}(\vec{X})$, *j* = 1, ⋯ , *k*_1_ *is a linear or non-linear function of* $\vec{X}$ *and* ${\text{feature}}_{k_{1}+1}(\vec{Z})$ *is a linear or non-linear function of* $\vec{Z}$ *and*

$$\hat{y}=\beta_{1}{\text{feature}}_{1}(\vec{X})+\cdots +\beta_{k_{1}}{\text{feature}}_{k_{1}}(\vec{X})+\beta_{k_{1}+1}\hat{t}+\beta_{k_{1}+2}{\text{feature}}_{k_{1}+1}(\vec{X},\hat{t})+\cdots +\;\;\beta_{k_{1}+k_{2}+1}{\text{feature}}_{k_{1}+k_{2}}(\vec{X},\hat{t})$$

*where each* ${\text{feature}}_{j}(\vec{X})$, *j* = 1, ⋯ , *k*_1_ *is a linear or non-linear function of* $\vec{X}$ *and* ${\text{feature}}_{j}(\vec{X},\hat{t})$, *j* = *k*_1_ + 1, ⋯ , *k*_1_ + *k*_2_ *is a non-linear function of* ($\vec{X}$, $\hat{t}$) *or a non-linear function of only* $\hat{t}$*, then then Constraint* 3 *(*$\beta^{T}X_{y}^{T}HX_{y}\beta -2\beta^{T}X_{y}^{T}H\vec{y}+{\vec{y}}^{T}H\vec{y}\leq \epsilon^{\prime }$*) is never active. This statement is true regardless of the dimension of* $\vec{X}$. *In other words, the following conditions are satisfied:*

1. the models in Stage One and Stage Two are general additive models that satisfy all the conditions in Theorem 2.
2. the model in Stage Two contains non-linear features of the predicted values of the treatment $\hat{t}$ and the covariates $\vec{X}$,

*then Constraint* 3 *(*$\beta^{T}X_{y}^{T}HX_{y}\beta -2\beta^{T}X_{y}^{T}H\vec{y}+{\vec{y}}^{T}H\vec{y}\leq \epsilon^{\prime }$*) is never active*.

**Proof**. Recall that Theorem 2 holds because of *H* = *H*_1_. In the Full version of Theorem 3, although *H* ≠ *H*_1_, *H* – *HH*_1_ = 0.

In Stage Two of our method, the optimal (minimum) solution of the objective function 2 ($\beta^{T}X_{y}^{T}X_{y}\beta -2\beta^{T}X_{y}^{T}\vec{y}+{\vec{y}}^{T}\vec{y}$) is ${\hat{\beta }}_{\text{min}}=[X_{y}^{T}X_{y}{]}^{-1}X_{y}^{T}\vec{y}$ and $\hat{y}=X_{y}{\hat{\beta }}_{\text{min}}=X_{y}[X_{y}^{T}X_{y}{]}^{-1}X_{y}^{T}\vec{y}=H_{1}\vec{y}$.

The objective function on the left side of Constraint 3 ($\beta^{T}X_{y}^{T}HX_{y}\beta -2\beta^{T}X_{y}^{T}H\vec{y}+{\vec{y}}^{T}H\vec{y}$) is:

$$\|H(\vec{y}-\hat{y}){\|}^{2}=\|H\vec{y}-H\hat{y}){\|}^{2}\;\;=\|H\vec{y}-HH_{1}y){\|}^{2}\;\text{where}\;\hat{y}=H_{1}y\;\;=\|(H-HH_{1})y){\|}^{2}\;\text{where}\;H-HH_{1}=0\;\;=0\leq \epsilon^{\prime }\;\text{where}\;\epsilon^{\prime }>0.$$

Therefore, Constraint 3 ($\beta^{T}X_{y}^{T}HX_{y}\beta -2\beta^{T}X_{y}^{T}H\vec{y}+{\vec{y}}^{T}H\vec{y}\leq \epsilon^{\prime }$) is always satisfied (i.e., never active).

**Next prove** *H* – *HH*_1_ = 0.

Recall the notation of the models in Stage One and Stage Two in the Full version of Theorem 3 and define the predictor matrices as follows:

Define the predictor matrix of *t* in Stage One:

$$X_{t}=({\text{feature}}_{1}(\vec{X}),\cdots ,{\text{feature}}_{k_{1}}(\vec{X}),{\text{feature}}_{k_{1}+1}(\vec{Z}){)}_{n\times (k_{1}+1)}\in R^{n\times (k_{1}+1)}$$

Define the predictor matrix of *y* in Stage Two:

$$X_{y}=({\text{feature}}_{1}(\vec{X}),\cdots ,{\text{feature}}_{k_{1}}(\vec{X}),\hat{t},{\text{feature}}_{k_{1}+1}(\vec{X},\hat{t}),\cdots ,{\text{feature}}_{k_{1}+k_{2}}(\vec{X},\hat{t}){)}_{n\times (k_{1}+k_{2}+1)}\;\;\in R^{n\times m}$$

where *m* = *k*_1_ + *k*_2_ + 1.

Next use the Gram-Schmidt algorithm to construct an orthogonal set of unit vectors.

Step 1: $u_{1}={\text{feature}}_{1}(\vec{X})$, $e_{1}=\frac{u_{1}}{|u_{1}|}$

Step 2: $u_{2}={\text{feature}}_{2}(\vec{X})-\frac{{\text{feature}}_{2}(\vec{X})\cdot u_{1}}{|u_{1}{|}^{2}}u_{1}$, $e_{2}=\frac{u_{2}}{|u_{2}|}$

.

.

.

Step *k*_1_ + 1: $u_{k_{1}+1}=\hat{t}-\frac{\hat{t}\cdot u_{1}}{|u_{1}{|}^{2}}u_{1}-\cdots -\frac{\hat{t}\cdot u_{k_{1}}}{|u_{k_{1}}{|}^{2}}u_{k_{1}}$, $e_{k_{1}+1}=\frac{u_{k_{1}+1}}{|u_{k_{1}+1}|}$

Step *k*_1_+2: $u_{k_{1}+2}={\text{feature}}_{k_{1}+1}(\vec{X},\hat{t})-\frac{{\text{feature}}_{k_{1}+1}(\vec{X},\hat{t})\cdot u_{1}}{|u_{1}{|}^{2}}u_{1}-\cdots -\frac{{\text{feature}}_{k_{1}+1}(\vec{X},\hat{t})\cdot u_{k_{1}+1}}{|u_{k_{1}+1}{|}^{2}}u_{k_{1}+1}$, $e_{k_{1}+2}=\frac{u_{k_{1}+2}}{|u_{k_{1}+2}|}$

.

.

.

Step *m*: $u_{m}={\text{feature}}_{k_{1}+k_{2}}(\vec{X},\hat{t})-\frac{{\text{feature}}_{k_{1}+k_{2}}(\vec{X},\hat{t})\cdot u_{1}}{|u_{1}{|}^{2}}u_{1}-\cdots -\frac{{\text{feature}}_{k_{1}+k_{2}}(\vec{X},\hat{t})\cdot u_{m-1}}{|u_{m-1}{|}^{2}}u_{m-1}$, $e_{m}=\frac{u_{m}}{|u_{m}|}$

Therefore the predictor matrix and t and y can be written as follows,

$$X_{t}=({\text{feature}}_{1}(\vec{X}),\cdots ,{\text{feature}}_{k_{1}}(\vec{X}),{\text{feature}}_{k_{1}+1}(\vec{Z}))\;\;=(u_{1},u_{2},\cdots ,u_{m}){\left(\begin{array}{cccc} 1 & a_{12} & \cdots & a_{1(k_{1}+1)}\\ 0 & 1 & \cdots & a_{2(k_{1}+1)}\\ \vdots & \vdots & & \vdots \\ 0 & 0 & \cdots & 1∕\omega_{k_{1}+1}\\ 0 & 0 & \cdots & 0\\ \vdots & \vdots & & \vdots \\ 0 & 0 & \cdots & 0 \end{array}\right)}_{m\times (k_{1}+1)}\;\;≕UA_{u}=(e_{1},e_{2},\cdots ,e_{m}){\left(\begin{array}{cccc} |u_{1}| & 0 & \cdots & 0\\ 0 & |u_{2}| & \cdots & 0\\ 0 & 0 & \cdots & 0\\ 0 & 0 & \cdots & |u_{m}| \end{array}\right)}_{m\times m}{\left(\begin{array}{cccc} 1 & a_{12} & \cdots & a_{1(k_{1}+1)}\\ 0 & 1 & \cdots & a_{2(k_{1}+1)}\\ \vdots & \vdots & & \vdots \\ 0 & 0 & \cdots & 1∕\omega_{k_{1}+1}\\ 0 & 0 & \cdots & 0\\ \vdots & \vdots & & \vdots \\ 0 & 0 & \cdots & 0 \end{array}\right)}_{m\times (k_{1}+1)}\;\;≕ED_{u}A_{u}\;\;≕EA$$

where diagonal entries of *D_u_* are *d_ii_* = ∣*u_i_*∣ > 0, and diagonal entries of *A_u_* are *a_ii_* = 1, 1 ≤ *i* ≤ *k*_1_ and *a*_(*k*_1_+1)(*k*_1_+1)_ = 1/*ω*_*k*_1_+1_. Therefore, the upper trapezoidal matrix *A* = *D_u_A_u_* has non-zero diagonal entries.

$$X_{y}=({\text{feature}}_{1}(\vec{X}),\cdots ,{\text{feature}}_{k_{1}}(\vec{X}),\hat{t},{\text{feature}}_{k_{1}+1}(\vec{X},\hat{t}),\cdots ,{\text{feature}}_{k_{1}+k_{2}}(\vec{X},\hat{t}))\;\;=(u_{1},u_{2},\cdots ,u_{m}){\left(\begin{array}{cccc} 1 & b_{12} & \cdots & b_{1m}\\ 0 & 1 & \cdots & b_{2m}\\ \vdots & \vdots & & \vdots \\ 0 & 0 & \cdots & 1 \end{array}\right)}_{m\times m}\;\;≕UB_{u}=(e_{1},e_{2},\cdots ,e_{m}){\left(\begin{array}{cccc} |u_{1}| & 0 & \cdots & 0\\ 0 & |u_{2}| & \cdots & 0\\ 0 & 0 & \cdots & 0\\ 0 & 0 & \cdots & |u_{m}| \end{array}\right)}_{m\times m}{\left(\begin{array}{cccc} 1 & b_{12} & \cdots & b_{1m}\\ 0 & 1 & \cdots & b_{2m}\\ \vdots & \vdots & & \vdots \\ 0 & 0 & \cdots & 1 \end{array}\right)}_{m\times m}\;\;≕ED_{u}B_{u}\;\;≕EB$$

where diagonal entries of *D_u_* are *d_ii_* = ∣*u_i_*∣ > 0, and diagonal entries of *B_u_* are *b_ii_* = 1, 1 ≤ *i* ≤ *m*. Therefore, the upper triangular matrix *B* = *D_u_B_u_* has non-zero diagonal entries.

The hat matrix *H* is

$$H=X_{t}[X_{t}^{T}X_{t}{]}^{-1}X_{t}^{T},\;\text{where}\;X_{t}=EA\;\;=EA[A^{T}E^{T}EA{]}^{-1}A^{T}E^{T},\;\text{where}\;E^{T}E=I_{m}\;\;=EA[A^{T}A{]}^{-1}A^{T}E^{T}\;\;=E\left(\begin{array}{cc} I_{k_{1}+1} & 0_{(k_{1}+1)\times k_{2}}\\ 0_{k_{2}\times (k_{1}+1)} & 0_{k_{2}} \end{array}\right)E^{T}.$$

The hat matrix *H*_1_ is

$$H_{1}=X_{y}[X_{y}^{T}X_{y}{]}^{-1}X_{y}^{T},\;\text{where}\;X_{y}=EB\;\;=EB[B^{T}E^{T}EB{]}^{-1}B^{T}E^{T},\;\text{where}\;E^{T}E=I_{m}\;\;=EB[B^{T}B{]}^{-1}B^{T}E^{T}\;\;=EI_{m}E^{T}.$$

Therefore,

$$HH_{1}=E\left(\begin{array}{cc} I_{k_{1}+1} & 0_{(k_{1}+1)\times k_{2}}\\ 0_{k_{2}\times (k_{1}+1)} & 0_{k_{2}} \end{array}\right)E^{T}EI_{m}E^{T},\;\text{where}\;E^{T}E=I_{m}\;\;=E\left(\begin{array}{cc} I_{k_{1}+1} & 0_{(k_{1}+1)\times k_{2}}\\ 0_{k_{2}\times (k_{1}+1)} & 0_{k_{2}} \end{array}\right)I_{m}E^{T}\;\;=E\left(\begin{array}{cc} I_{k_{1}+1} & 0_{(k_{1}+1)\times k_{2}}\\ 0_{k_{2}\times (k_{1}+1)} & 0_{k_{2}} \end{array}\right)E^{T}\;\;=H.$$

## Appendix C. Tables

**Table 9:** ID instrument, results using two-stage methods

Index	Data Generation	Error Term	Predictor Matrices	Fraction of time that the constraint holds / Fraction of time that the left side of the constraint is zero
Row 1-3 state that the constraint is always satisfied (never active) regardless of the type of the error term when models in both stages are linear models and the data generation and prediction models share the same model forms.				
1	*t* = *x* + *z* + *e*_1_ *y* = *x* + *t* + *e*_2_	Gaussian error	*X_t_* = (*x*, *z*) *X_y_* = (*x*, $\hat{t}$)	100% / 100%
2	*t* = *x* + *z* + *e*_1_ *y* = *x* + *t* + *e*_2_	mixture error	*X_t_* = (*x*, *z*) *X_y_* = (*x*, $\hat{t}$)	100% / 100%
3	*t* = *x* + *z* + *e*_1_ *y* = *x* + *t* + *e*_2_	fanning error	*X_t_* = (*x*, *z*) *X_y_* = (*x*, $\hat{t}$)	100% / 100%
Row 4-6 state that the constraint is always satisfied (never active) regardless of the type of the error term when the model in the first stage is linear, the model in the second stage is a general additive model, and the data generation and prediction models share the same model forms.				
4	*t* = *x* + *z* + *e*_1_ *y* = *x* + *t* + *t*^2^ + *e*_2_	Gaussian error	*X_t_* = (*x*, *z*) *X_y_* = (*x*, $\hat{t}$, ${\hat{t}}^{2}$)	100% / 100%
5	*t* = *x* + *z* + *e*_1_ *y* = *x* + *t* + *t*^2^ + *e*_2_	mixture error	*X_t_* = (*x*, *z*) *X_y_* = (*x*, $\hat{t}$, ${\hat{t}}^{2}$)	100% / 100%
6	*t* = *x* + *z* + *e*_1_ *y* = *x* + *t* + *t*^2^ + *e*_2_	fanning error	*X_t_* = (*x*, *z*) *X_y_* = (*x*, $\hat{t}$, ${\hat{t}}^{2}$)	100% / 100%
Row 7-10 state that the constraint is always satisfied (never active) even when the data generation and prediction models do not have the same model forms.				
7	*t* = *x*^2^ + *z*^2^ + *e*_1_ *y* = *x*^2^ + *t*^2^ + *e*_2_	fanning error	*X_t_* = (*x*, *z*) *X_y_* = (*x*, $\hat{t}$)	100% / 100%
8	*t* = sin(*x*) + cos(*z*) + *e*_1_ *y* = cos(*x*) + sin(*t*) + *e*_2_	fanning error	*X_t_* = (*x*, *z*) *X_y_* = (*x*, $\hat{t}$)	100% / 100%
9	*t* = *x*^2^ + *z*^2^ + *e*_1_ *y* = *x*^2^ + *t*^2^ + *e*_2_	fanning error	*X_t_* = (*x*, *z*) *X_y_* = (*x*, $\hat{t}$, ${\hat{t}}^{2}$, $x\hat{t}$)	100% / 100%
10	*t* = sin(*x*) + cos(*z*) + *e*_1_ *y* = cos(*x*) + sin(*t*) + *e*_2_	fanning error	*X_t_* = (*x*, *z*) *X_y_* = (*x*, $\hat{t}$, ${\hat{t}}^{2}$, $x\hat{t}$)	100% / 100%

**Table 10:** 2+-D instrument: results using two-stage methods. Sum of fifth and sixth columns is always 100%.

Index	Data Generation	Predictor Matrices	Epsilon Percent *γ*	Fraction of cases when constraint holds	Fraction of cases with no feasible solution	Total Fraction
Row 1-3 state that in the multi-dimensional cases for instrument *z*, either the constraint is not active or there is no feasible solution when models in both stage are linear and the data generation and prediction models share the same model forms.						
1	*t* = *x* + *z*_1_ + *z*_2_ + *e*_1_ *y* = *x* + *t* + *e*_2_	*X_t_* = (*x*, *z*_1_, *z*_2_) *X_y_* = (*x*, $\hat{t}$)	0.5%	99.9%	0.1%	100%
2	*t* = *x* + *z*_1_ + *z*_2_ + *e*_1_ *y* = *x* + *t* + *e*_2_	*X_t_* = (*x*, *z*_1_, *z*_2_) *X_y_* = (*x*, $\hat{t}$)	0.1%	84%	16%	100%
3	*t* = *x*_1_ + *x*_2_ + *z*_1_+ *z*_2_ + *z*_3_ + *e*_1_ *y* = *x*_1_ + *x*_2_ + *t* + *e*_2_	*X_t_* = (*x*_1_, *x*_2_, *z*_1_, *z*_2_, *z*_3_) *X_y_* = (*x*_1_, *x*_2_, $\hat{t}$)	0.5%	99.5%	0.5%	100%
Row 4-6 state that in the multi-dimensional cases for instrument *z*, either the constraint is not active or there is no feasible solution when the model in the first stage is a general additive model, the model in the second stage is linear and the data generation and prediction models share the same model forms.						
4	*t* = *x*_1_ + *x*_2_ + *z*_1_ + *z*_2_ + *z*_3_ + *e*_1_ *y* = *x*_1_ + *x*_2_ + *t* + *e*_2_	*X_t_* = (*x*_1_, *x*_2_, *z*_1_, *z*_2_, *z*_3_) *X_y_* = (*x*_1_, *x*_2_, $\hat{t}$)	0.1%	64.8%	35.2%	100%
5	$t=x_{1}+x_{2}+z_{1}+z_{2}+x_{1}^{2}+x_{2}^{2}+z_{1}^{2}+z_{2}^{2}+e_{1}$ *y* = *x*_1_ + *x*_2_ + *t* + *e*_2_	$X_{t}=(x_{1},x_{2},z_{1},z_{2},x_{1}^{2},x_{2}^{2},z_{1}^{2},z_{2}^{2})$ $X_{y}=(x_{1},x_{2},\hat{t})$	0.5%	84.8%	15.2%	100%
6	*t* = *x*_1_ + *x*_2_ + *z*_1_ + *z*_2_ + *x*_1_*z*_1_ + *x*_2_*z*_2_ + *e*_1_ *y* = *x*_1_ + *x*_2_ + *t* + *e*_2_	*X_t_* = (*x*_1_, *x*_2_, *z*_1_, *z*_2_, *x*_1_*z*_1_, *x*_2_*z*_2_) *X_y_* = (*x*_1_, *x*_2_, $\hat{t}$)	0.5%	96.0%	4.0%	100%
Row 7-10 state that in the multi-dimensional cases for instrument *z*, either the constraint is not active or there is no feasible solution even when the data generation and prediction models do not have the same model forms.						
7	$t=x_{1}^{2}+x_{2}^{2}+z_{1}^{2}+z_{2}^{2}+e_{1}$ $y=x_{1}^{2}+x_{2}^{2}+t^{2}+e_{2}$	*X_t_* = (*x*_1_, *x*_2_, *z*_1_, *z*_2_) *X_y_* = (*x*_1_, *x*_2_, $\hat{t}$)	0.1%	61.6%	38.4%	100%
8	*t* = sin(*x*_1_) + sin(*x*_2_) + cos(*z*_1_) + cos(*z*_2_) + *e*_1_ *y* = sin(*x*_1_) + sin(*x*_2_) + cos(*t*) + *e*_2_	*X_t_* = (*x*_1_, *x*_2_, *z*_1_, *z*_2_) *X_y_* = (*x*_1_, *x*_2_, $\hat{t}$)	0.1%	71.7%	28.3%	100%
9	$t=x_{1}^{2}+x_{2}^{2}+z_{1}^{2}+z_{2}^{2}+e_{1}$ $y=x_{1}^{2}+x_{2}^{2}+t^{2}+e_{2}$	*X_t_* = (*x*_1_, *x*_2_, *z*_1_, *z*_2_, *x*_1_*z*_1_, *x*_2_*z*_2_) *X_y_* = (*x*_1_, *x*_2_, $\hat{t}$)	0.5%	97.8%	2.0%	100%
10	*t* = sin(*x*_1_) + sin(*x*_2_) + cos(*z*_1_) + cos(*z*_2_) + *e*_1_ *y* = sin(*x*_1_) + sin(*x*_2_) + cos(*t*) + *e*_2_	*X_t_* = (*x*_1_, *x*_2_, *z*_1_, *z*_2_, *x*_1_*z*_1_, *x*_2_*z*_2_) *X_y_* = (*x*_1_, *x*_2_, $\hat{t}$)	0.5%	90.9%	9.1%	100%

**Table 11:** Results using Our One-Stage Method. The sum of percentages, which is always 100%, is the fourth and second last columns, plus the maximum of the fifth and sixth columns.

Index	Data Generation	Predictor Matrices	Fraction of cases that constraint holds	Fraction of cases with a feasible solution for the two- stage method	Fraction of cases with a feasible solution for the one- stage method	Fraction of cases with no feasible solution	Total Fraction
Row 1-4 state that the fractions of time that there is a feasible solution are the same for our two-stage and one-stage method when models in both stages are general additive models.							
1	*t* = *x* + *z* + *xz* + *e*_1_ *y* = *x* + *t* + *xt* + *e*_2_	*X_t_* = (*x*, *z*, *xz*) *X_y_* = (*x*, $\hat{t}$, $x\hat{t}$)	99.0%	1.0%	1.0%	0.0%	100%
2	*t* = *x* + *z* + *z*^2^ + *e*_1_ *y* = *x* + *t* + *t*^2^ + *e*_2_	*X_t_* = (*x*, *z*, *z*^2^) *X_y_* = (*x*, $\hat{t}$, ${\hat{t}}^{2}$)	97.0%	3.0%	3.0%	0.0%	100%
3	*t* = *x* + *x*^2^ + *z* + *z*^2^ + *e*_1_ *y* = *x* + *x*^2^ + *t* + *t*^2^ + *e*_2_	*X_t_* = (*x*, *z*, *z*^2^) *X_y_* = (*x*, $\hat{t}$, ${\hat{t}}^{2}$)	96.0%	4.0%	4.0%	0.0%	100%
4	$t=x+z_{1}+z_{2}+z_{1}^{2}+z_{2}^{2}+e_{1}$ *y* = *x*+*t*+*t*^2^+*e*_2_	*X_t_* = (*x*, *z*_1_, *z*_2_, $z_{1}^{2}$, $z_{2}^{2}$) *X_y_* = (*x*, $\hat{t}$, ${\hat{t}}^{2}$)	98.0%	1.0%	1.0%	1.0%	100%
Row 5-8 state that the fraction of time that there is a feasible solution for our one-stage method are greater than that for two-stage method when the model in the first stage is a linear or general additive model and the model in the second stage is linear.							
5	*t* = *x* + *z* + *xz* + *e*1 *y* = *x* + *t* + *e*_2_	*X_t_* = (*x*, *z*, *xz*) *X_y_* = (*x*, $\hat{t}$)	92.0%	0.0%	8.0%	0.0%	100%
6	*t* = *x* + *z* + *z*^2^ + *e*_1_ *y* = *x* + *t* + *e*_2_	*X_t_* = (*x*, *z*, *z*^2^) *X_y_* = (*x*, $\hat{t}$)	96.0%	0.0%	4.0%	0.0%	100%
7	*t* = *x* + *x*^2^ + *z* + *z*^2^ + *e*_1_ *y* = *x* + *t* + *e*_2_	*X_t_* = (*x*, *z*, *xz*) *X_y_* = (*x*, $\hat{t}$)	88.0%	0.0%	12.0%	0.0%	100%
8	*t* = *x* + *z*_1_ + *z*_2_ + *e*_1_ *y* = *x* + *t* + *e*_2_	*X_y_* = (*x*, *z*_1_, *z*_2_) *X_y_* = (*x*, $\hat{t}$)	96%	0.0%	2.0%	2.0%	100%

## Appendix D. Additional details of the two stage approach

### Vectorized Version with General Loss

All quantities are vectorized in this version.

#### Stage One

$$\omega \in \text{arg}\;\underset{\omega }{\text{min}}\;loss(\vec{t},\hat{t})\;\text{where}\;\hat{t}=f_{\omega }(\vec{X},\vec{Z})$$

#### Stage Two

$$\;\beta \in \text{arg}\;\underset{\beta }{\text{min}}\;loss(\vec{y},\hat{y})\;\text{where}\;\hat{y}=g_{\beta }(\vec{X},\hat{t})\;\;\text{s.t.}\;\;\;\beta \;\;\;\text{obeys}\;loss(\vec{r},\hat{r})\geq loss(\vec{r},0)-\epsilon^{\prime }\;\text{where}\;\;\;\vec{r}\;=\vec{y}-\hat{y}\;\;\text{and}\;\;\;\hat{r}\;=h_{\alpha }(\vec{X},\vec{Z}),\;\text{where}\;\alpha \in \text{arg}\;\underset{\alpha }{\text{min}}\;loss(\vec{r},\hat{r}).$$

When the squared loss is chosen as the loss function, many of the calculations simplify, allowing us to gain insight into the structure of the problem and its solution. Thus, we state the formulation above with the squared loss, and then simplify it.

### Vectorized Version with Squared Loss

In what follows, we replaced the general loss function with the squared loss function.

#### Stage One

$$\omega \in \text{arg}\;\underset{\omega }{\text{min}}\;(\vec{t}-\hat{t}{)}^{T}(\vec{t}-\hat{t})\;\text{where}\;\hat{t}=f_{\omega }(\vec{X},\vec{Z})$$

#### Stage Two

$$\;\beta \in \text{arg}\;\underset{\beta }{\text{min}}\;(\vec{y}-\hat{y}{)}^{T}(\vec{y}-\hat{y})\;\text{where}\;\hat{y}=g_{\beta }(\vec{X},\hat{t})\;\;\text{s.t.}\;\;\;\beta \;\;\;\text{obeys}\;(\vec{r}-\hat{r}{)}^{T}(\vec{r}-\hat{r})\geq (\vec{r}-0{)}^{T}(\vec{r}-0)-\epsilon^{\prime }\;\text{where}\;\;\;\vec{r}\;=\vec{y}-\hat{y}\;\;\text{and}\;\;\;\hat{r}\;=h_{\alpha }(\vec{X},\vec{Z}),\;\text{where}\;\alpha \in \text{arg}\;\underset{\alpha }{\text{min}}\;(\vec{r}-\hat{r}{)}^{T}(\vec{r}-\hat{r}).$$

### Simplified Version

Let us define $X_{t}\;=\;X_{t}(\vec{X},\vec{Z})$, $X_{y}\;=\;X_{y}(\vec{X},\hat{t})$, and $X_{r}\;=\;X_{r}(\vec{X},\vec{Z})$ to be the predictor matrices of $\vec{t}$, $\vec{y}$, and $\vec{r}$ respectively. Also, $\hat{t}=X_{t}\hat{\omega }$ and $\hat{\omega }$ is the result of Stage One.

Then, let us solve for *α* using the squared loss functions, so that $\alpha \;=\;(X_{t}^{T}X_{t}{)}^{-1}X_{t}^{T}\vec{r}$ where $\vec{r}=\vec{y}-\hat{y}$ and $\hat{y}=X_{y}\beta$. We will use this to eliminate *α* in the following version of the problem.

#### Stage One

(1)
$$\omega \in \text{arg}\;\underset{\omega }{\text{min}}\;\omega^{T}X_{t}^{T}X_{t}\omega -2\omega^{T}X_{t}^{T}\vec{t}+{\vec{t}}^{T}\vec{t}$$

#### Stage Two

(2)
$$\beta \in \text{arg}\;\underset{\beta }{\text{min}}\;\beta^{T}X_{y}^{T}X_{y}\beta -2\beta^{T}X_{y}^{T}\vec{y}+{\vec{y}}^{T}\vec{y}$$

(3)
$$\text{s.t.}\;\;\;\beta \;\;\;\text{obeys}\;\beta^{T}X_{y}^{T}HX_{y}\beta -2\beta^{T}X_{y}^{T}H\vec{y}+{\vec{y}}^{T}H\vec{y}\leq \epsilon^{\prime }$$

where $\epsilon^{\prime }\;=\;\gamma {\tilde{r}}^{T}\tilde{r}$, and $\tilde{r}$ is obtained by the traditional two stage method without constraints, i.e.,

$$\tilde{r}=\vec{y}-H_{1}\vec{y}=\vec{y}-X_{y}(\vec{X},H\vec{t})(X_{y}(\vec{X},H\vec{t}{)}^{T}X_{y}(\vec{X},H\vec{t}){)}^{-1}X_{y}(\vec{X},H\vec{t}){)}^{T}\vec{y},$$

where $H\;=\;X_{r}(X_{r}^{T}X_{r}{)}^{-1}X_{r}^{T}$ and $H_{1}\;=\;X_{y}(X_{y}^{T}X_{y}{)}^{-1}X_{y}^{T}$.

The first stage is the same as that of the traditional two stage method when *f_ω_* is a linear model with coefficients *ω*, and squared loss. The second stage has a model for outcomes $\hat{y}$ depending on $\hat{t}$ and the covariates $\vec{X}$ as the traditional method does, however, the model is constrained by an ellipsoidal constraint. The constraint says that if we were to use the instrument to model the remainder (generating a model $\hat{r}$ to fit $\vec{r}$) then despite our efforts, we cannot predict the remainder much better than if we had used a model that was identically 0.

At this point, we have derived the final simplified two stage least squares model with the new machine learning constraint for the general additive case. This can be directly implemented according to the formulas above. Although there is not a closed-form solution for other losses, the formulations can also be applied in practice, assuming access to optimization methods that can handle the new objectives and constraint. Now we will switch over to the one stage method.

## Appendix E. Additional details on the one stage optimization approach

### Vectorized Version with General Loss

All quantities are vectorized in this version.

$$\;\underset{\beta ,\omega }{\text{min}}\;loos(\vec{y},\hat{y})\;\text{where}\;\hat{y}=g_{\beta }(\vec{X},\hat{t}),\;\text{and}\;\hat{t}=f_{\omega }(\vec{X},\vec{Z})\;\;\text{s.t.}\;\;\omega \;\;\text{obeys}\;loss(\vec{t},\hat{t})\leq loss(\vec{t},f_{\omega }(\vec{X}))-\epsilon \;\;\text{and}\;\;\beta \;\;\text{obeys}\;loss(\vec{r},\hat{r})\geq loss(\vec{r},0)-\epsilon^{\prime }\;\text{where}\;\;\vec{r}\;=\vec{y}=\hat{y}\;\;\text{and}\;\;\hat{r}\;=h_{\alpha }(\vec{X},\vec{Z}),\;\text{where}\;\alpha \in \text{arg}\underset{\alpha }{\text{min}}loss(\vec{r},\hat{r}).$$

### Vectorized Version with Squared Loss

Again we work with the squared loss, which allows for simplification.

$$\;\underset{\beta ,\omega }{\text{min}}\;(\vec{y}-\hat{y}{)}^{T}(\vec{y}-\hat{y})\;\text{where}\;\hat{y}=g_{\beta }(\vec{X},\hat{t}),\;\text{and}\;\hat{t}=f_{\omega }(\vec{X},\vec{Z})\;\;\text{s.t.}\;\;\omega \;\;\text{obeys}\;(\vec{t}-\hat{t}{)}^{T}(\vec{t}-\hat{t})\leq (\vec{t}-f_{\omega }(\vec{X}){)}^{T}(\vec{t}-f_{\omega }(\vec{X}))-\epsilon \;\;\text{and}\;\;\beta \;\;\text{obeys}\;(\vec{r}-\hat{r}{)}^{T}(\vec{r}-\hat{r})\geq (\vec{r},0{)}^{T}(\vec{r}-0)-\epsilon^{\prime }\;\text{where}\;\;\vec{r}\;=\vec{y}=\hat{y}\;\;\text{and}\;\;\hat{r}\;=h_{\alpha }(\vec{X},\vec{Z}),\;\text{where}\;\alpha \in \text{arg}\underset{\alpha }{\text{min}}(\vec{r}-\hat{r}{)}^{T}(\vec{r}-\hat{r}).$$

### Simplified Version

Let us define $X_{t}\;=\;X_{t}(\vec{X},\vec{Z})$ and $X_{y}\;=\;X_{y}(\vec{X},X_{t}\omega )$ to be the predictor matrices of $\vec{t}$ and $\vec{y}$ respectively.

Then, let us solve for *α* using the squared loss functions, so that $\alpha \;=\;(X_{t}^{T}X_{t}{)}^{-1}X_{t}^{T}\vec{r}$ where $\vec{r}=\vec{y}-\hat{y}$ and $\hat{y}=X_{y}\beta$. We will use this to eliminate *α* in the following version of the problem.

(3)
$$\underset{\beta ,\omega }{\text{min}}\;\beta^{T}X_{y}^{T}X_{y}\beta -2\beta^{T}X_{y}^{T}\vec{y}+{\vec{y}}^{T}\vec{y}$$

(5)
$$\text{s.t.}\;\;\;\omega \;\;\;\text{obeys}\;\omega^{T}X_{t}^{T}X_{t}\omega -2\omega^{T}X_{t}^{T}\vec{t}+{\vec{t}}^{T}\vec{t}\leq \epsilon$$

(6)
$$\text{and}\;\;\;\beta \;\;\;\text{obeys}\;\beta^{T}X_{y}^{T}HX_{y}\beta -2\beta^{T}X_{y}^{T}H\vec{y}+{\vec{y}}^{T}H\vec{y}\leq \epsilon^{\prime }.$$

Here, $\epsilon =\gamma {\vec{t}}^{T}\vec{t}$, $\epsilon^{\prime }=\gamma {\tilde{r}}^{T}\tilde{r}$, and $H\;=\;X_{r}(X_{r}^{T}X_{r}{)}^{-1}X_{r}^{T}$. Also $\tilde{r}$ is obtained by the traditional two stage method without constraints i.e.

$$\tilde{r}=\vec{y}-X_{y}(X_{y}^{T}X_{y}{)}^{-1}X_{y}^{T}\vec{y}.$$
